# Subgenome Dominance in Allotetraploid *Actinidia valvata* Regulates RNA m^6^A Modification for Waterlogging Tolerance

**DOI:** 10.1002/advs.202503974

**Published:** 2025-06-05

**Authors:** Xiaoli Hu, Changbin Xu, Xiaolan Li, Lin Li, Yu Bao, Miaofeng Gu, Xinyi Li, Liuqing Huo, Jinli Gong, Xiaolong Li, Minhui Wang, Kai Xu, Xueren Yin, Zhangjun Fei, Xuepeng Sun

**Affiliations:** ^1^ National Key Laboratory for Development and Utilization of Forest Food Resources Zhejiang A&F University Hangzhou Zhejiang 311300 China; ^2^ Key Laboratory of Quality and Safety Control for Subtropical Fruit and Vegetable Ministry of Agriculture and Rural Affairs Zhejiang A&F University Hangzhou Zhejiang 311300 China; ^3^ School of Horticulture Anhui Agricultural University Hefei 230036 China; ^4^ Boyce Thompson Institute Cornell University Ithaca NY 14853 USA

**Keywords:** actinidia, plant polyploid, post‐transcriptional modification, subgenome dominance

## Abstract

Polyploidization is a major driving force for evolutionary innovation and environmental adaptation in plants and is notably prevalent in kiwifruits. However, the molecular mechanisms through which subgenome interactions influence vigor and stress resilience remain largely unclear. Here, the high‐quality genome of the tetraploid kiwifruit *Actinidia valvata*, which exhibits strong waterlogging tolerance compared to cultivated varieties is reported. The analysis reveals that the polyploid genome is of hybrid origin and exhibits subgenome dominance. The enhanced gene expression in the dominant subgenome is accompanied by fewer transposable elements, lower DNA methylation levels, increased chromatin accessibility, and biased global RNA m^6^A methylation abundance and distribution. It is demonstrated that this dominance is established, in part, prior to polyploidization. The dominant subgenome is transcriptionally more responsive to waterlogging stress, consistent with the fact that its putative progenitor is also waterlogging tolerant, affirming the significant role of the dominant subgenome in mediating waterlogging tolerance in *A. valvata*. Furthermore, inhibition of RNA m^6^A methylation in *A. valvata* roots enhances their activity under waterlogging stress, while waterlogging modulates m^6^A modifications, particularly in the dominant subgenome, affecting genes known to be involved in waterlogging responses. These findings reveal that subgenome dominance in *A*. *valvata* operates through multiple regulatory mechanisms, collectively endowing the polyploid with unique traits inherited from its progenitors.

## Graphical abstract

The waterlogging tolerance of allotetraploid *Actinidia valvata* is inherited from one of its diploid progenitors, which leads to subgenome expression dominance, mediated by complex mechanisms including altered chromatin accessibility and DNA methylation. Waterlogging treatment triggers upregulation of key stress‐responsive genes, partly through dynamic regulation of RNA m^6^A modifications.

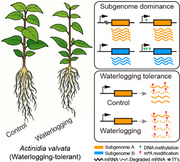

## 1 Introduction

Polyploidization, the multiplication of chromosomes, creates genomic conditions that can drive evolutionary innovation in both plants and animals.^[^ ^1^, ^2^ ^]^ This process can enhance genetic diversity at the genomic,^[^ ^2^, ^3^ ^]^ transcriptomic,^[^ ^4^, ^5^ ^]^ and epigenomic^[^ ^6^, ^7^, ^8^ ^]^ levels, offering polyploids a competitive advantage in diverse and extreme environments. Polyploids include allopolyploids, such as wheat,^[^ ^9^ ^]^ cotton,^[^ ^10^ ^]^ Brassica,^[^ ^11^ ^]^ and oat,^[^ ^12^ ^]^ as well as autopolyploids, like potatoes,^[^ ^13^ ^]^ alfalfa,^[^ ^14^ ^]^ and sugarcane.^[^ ^15^ ^]^ Allopolyploidy combines genetic material from different species, potentially resulting in more resilient offspring that exhibit distinct morphological and physiological traits compared to their diploid progenitors, such as larger cells, modified leaf shapes, bigger flowers and fruits, and sometimes increased stress tolerance and vigor.^[^ ^16^ ^]^ Many allopolyploids exhibit subgenome dominance, where one set of chromosomes has more dominant gene expression than the others,^[^ ^17^, ^18^ ^]^ ultimately influencing trait inheritance. However, the molecular mechanisms by which subgenome interactions influence morphological traits remain largely unclear in most polyploids.

Kiwifruit is a perennial and dioecious climbing woody vine belonging to the genus *Actinidia*. Over 50 species of *Actinidia* have been recognized worldwide, with most native to China.^[^ ^19^ ^]^ Polyploidy is prevalent in *Actinidia*, and species exhibit a range of ploidy levels, from diploid to octoploid.^[^ ^20^ ^]^ Even within the same species, different ploidy levels can be observed. For example, both diploid and polyploid genotypes exist in the primary cultivars of kiwifruit, the yellow‐fleshed *A*. *chinensis* (mainly diploids)^[^ ^21^ ^]^ and the green‐fleshed *A*. *deliciosa* (mainly hexaploids).^[^ ^22^ ^]^ The high frequency of polyploids in the natural population of *Actinidia* is likely due to the ability to produce a relatively high proportion of unreduced (2n) gametes during meiosis,^[^ ^23^ ^]^ which makes kiwifruit an excellent system for studying the causes and consequences of polyploidization.

Kiwifruit plants are sensitive to waterlogging, which is increasingly problematic as global cultivation demands adaptation to new environments that may frequently experience heavy rainfall. In plants, responses to waterlogging are regulated by a variety of spatiotemporal signals,^[^ ^24^ ^]^ primarily involving hypoxia signaling induced by waterlogging and the accumulation of ethylene.^[^ ^25^ ^]^ These signals modulate adaptive root growth^[^ ^26^ ^]^ and hypoxia stress acclimation responses, including metabolic shifts to maintain ATP production through enhanced anaerobic metabolism and mitigate reactive oxygen species (ROS), while simultaneously reducing energy‐demanding biological processes.^[^ ^27^ ^]^ In kiwifruit, various morphological and physiological features make the plants particularly vulnerable to low oxygen levels in the root zone under waterlogged conditions. Specifically, kiwifruit vines lack the ability to develop aerenchyma tissue in their roots, a feature that typically helps plants adapt to low oxygen conditions,^[^ ^28^ ^]^ although the gene regulatory network mediated by ERF‐VII transcription factors in response to waterlogging appears to be generally conserved.^[^ ^29^, ^30^, ^31^ ^]^ Grafting elite cultivar scions onto waterlogging‐tolerant rootstocks has proven to be an effective strategy for improving waterlogging tolerance in crops and fruit trees.^[^ ^32^, ^33^ ^]^ Previous screening of *Actinidia* germplasm identified *A*. *macrosperma* and *A*. *valvata* as having high waterlogging tolerance compared to cultivated kiwifruits.^[^ ^34^, ^35^ ^]^ *A*. *valvata* is a tetraploid^[^ ^36^ ^]^ and has increasingly been used as a rootstock for commercial kiwifruit cultivars. However, the nature of the polyploidization and the mechanism of waterlogging tolerance in *A*. *valvata* remain elusive.

N^6^‐methyladenosine (m^6^A), the most prevalent RNA modification in eukaryotes, undergoes dynamic regulation through an evolutionarily conserved system comprising methyltransferases (writers), demethylases (erasers) and recognition proteins (readers). Growing evidence highlights m^6^A as a central regulatory mechanism in plant mRNA metabolism during stress adaptation. Notably, m^6^A modification influences plant‐pathogen interactions, with studies linking it to enhanced powdery mildew resistance in apple and impaired infectivity of *Magnaporthe oryzae* in rice.^[^ ^37^, ^38^ ^]^ In *Arabidopsis*, m^6^A marks on transcripts encoding salt and osmotic stress response proteins enhance their stability by suppressing site‐specific ribonucleolytic cleavage under salt stress, thereby promoting salt tolerance.^[^ ^39^ ^]^ Systematic studies have further mapped stress‐specific m^6^A methylomes and their coordination with transcriptomic changes in diverse plant species under environmental stress, including heat‐stressed pak‐choi seedlings,^[^ ^40^ ^]^ cold‐exposed tomato anthers,^[^ ^41^ ^]^ drought‐treated apple leaves,^[^ ^42^ ^]^ and salt‐challenged sorghum.^[^ ^43^ ^]^ Although m^6^A‐related genes have been identified in kiwifruit,^[^ ^44^ ^]^ their functional role and regulatory mechanisms in stress responses remain unexplored.

In this study, we aimed to uncover the mechanisms underlying waterlogging tolerance in the tetraploid kiwifruit *A*. *valvata* (2n = 4x = 116) (Figure S1, Supporting Information). To achieve this, we assembled a chromosome‐scale reference genome for *A*. *valvata*. Through integrative analyses of gene expression, chromatin accessibility, DNA methylation, and RNA m^6^A modification, we gained a detailed understanding of the mechanisms driving subgenome dominance in *A*. *valvata* and demonstrated the regulatory role of m^6^A modification in mediating waterlogging responses. These findings provide a foundation for understanding the evolutionary mechanisms, species differentiation, and environmental adaptation of kiwifruit species and will contribute to kiwifruit research and breeding.

## 2 Results

### 2.1 Genome Assembly and Annotation of the Tetraploid Kiwifruit *Actinidia valvata*

**Figure 1:**
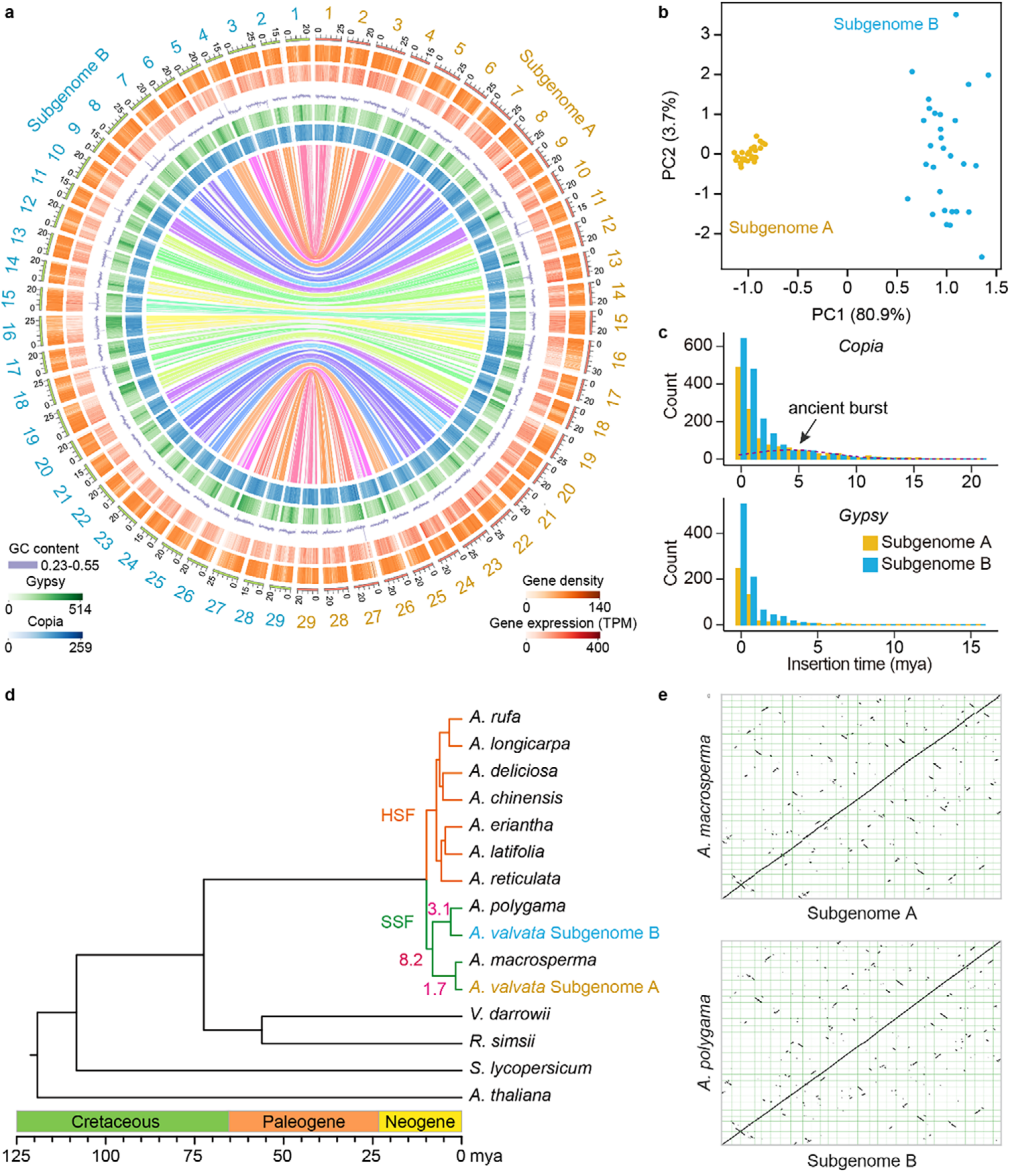
Genome assembly of tetraploid *A. valvata*. a) Circos plot of the *A. valvata* genome. The outermost track displays the chromosomes of the two subgenomes, measured in megabases (Mb). Moving inward, the subsequent tracks illustrate summarized statistics based on 100 kb sliding windows: gene density, gene expression, GC content, *Gypsy* content, and *Copia* content. Colored lines connecting different chromosomes represent syntenic relationships. b) PCA analysis of *A. valvata* chromosomes based on the statistics of transposable elements and unique *k*‐mers. c) Age distribution of *Copia* and *Gypsy* families of LTR‐RTs. d) Divergence times of 14 representative species. Colored branches indicate two different groups of kiwifruits, hairy‐skinned fruit (HSF) and smooth‐skinned fruit (SSF). Numbers on the branches indicate divergence times in million years ago (mya). The maximum likelihood phylogeny of species was constructed using low‐copy orthogroups. e) Genome collinearity between the two subgenomes and their closest ancestors, *A*. *macrosperma* and *A*. *polygama*, respectively.

We assembled the *A. valvata* genome using 23.6 Gb of PacBio HiFi sequences (Table S1, Supporting Information), resulting in a draft primary assembly comprising 3994 contigs with a cumulative size of 1.79 Gb and an N50 length of 1.12 Mb. The tetraploid nature of the genome complicated haplotig purging; however, principal component analysis (PCA) of the assembly based on repetitive *k‐*mers^[^ ^45^ ^]^ clearly separated the contigs into two groups, representing two distinct subgenomes. Therefore, haplotigs within each group were purged separately, after which all nonredundant contigs were pooled together for error correction with 138.4 Gb of MGI short‐read sequences (≈106.4× coverage) and subsequently for scaffolding with 186.2 Gb of Hi‐C sequences (≈143.2× coverage). The final assembly had a size of 1.36 Gb with a contig N50 of 1.42 Mb (Table S2, Supporting Information), among which 98.4% were anchored to 58 chromosomes (**Figure** **1**a). Consequently, two subgenomes were delineated from the assembly according to PCA analysis (Figure 1b). Subgenome A was determined to be 695.8 Mb, larger than subgenome B (645.3 Mb) (Table S2, Supporting Information).

The quality of the genome assembly was assessed using multiple approaches. First, the Hi‐C map demonstrated well‐organized chromatin interactions along the diagonals (Figure S2, Supporting Information), and genome comparison revealed an overall high collinearity with the published *A*. *chinensis* genome (Figure S3, Supporting Information).^[^ ^46^ ^]^ Alignment of short reads against the assembly revealed that 99.0% of genomic regions could be covered by at least one read. The LTR assembly index (LAI) score of the assembly was 12.92, exceeding the standard for a reference genome,^[^ ^47^ ^]^ and BUSCO^[^ ^48^ ^]^ evaluation indicated that 99.2% of plant conserved single‐copy orthologs were fully captured by the *A*. *valvata* assembly (Table S2, Supporting Information).

We annotated a total of 645 Mb of repetitive sequences, accounting for 47.28% of the assembled *A. valvata* genome (Table S3, Supporting Information). Long terminal repeat retrotransposons (LTR‐RTs), particularly those from the *Copia* (10.08%) and *Gypsy* (9.34%) families, were the most abundant. Notably, *Gypsy* LTR‐RTs were predominantly found in centromeric regions, whereas *Copia* LTR‐RTs were widely dispersed throughout the chromosomes (Figure S4, Supporting Information). The estimated insertion times suggested an ancient LTR‐RT expansion (≈5 MYA) in the *Copia* family, likely resulting in its prevalence over *Gypsy* LTR‐RTs (Figure 1c). This pattern contrasts with that observed in many other plants.^[^ ^49^ ^]^ LTR‐RTs can be eliminated through unequal recombination, leading to the formation of solo‐LTRs in the genome. The ratios of solo to intact LTRs were 2.7 in subgenome A and 3.7 in subgenome B, indicating a more rapid turnover of LTR‐RTs in subgenome B, potentially contributing to its smaller size. By integrating homology‐ and RNA‐Seq based methods, we predicted a total of 90 251 protein‐coding genes within the *A*. *valvata* genome, with the majority (89.7%) being functionally annotated. RNA‐Seq read alignment against the assembly showed a mapping rate of 96.7%. These results collectively underscore the high quality of the *A*. *valvata* genome assembly and annotation.

### 2.2 Allotetraploid Origin of the *A. valvata* Genome

More than 50 species have been described in the genus *Actinidia*, which can be categorized into two monophyletic groups characterized by their hairy‐skinned fruit (HSF) or smooth‐skinned fruit (SSF).^[^ ^50^, ^51^ ^]^ *A*. *valvata* produces smooth‐skinned fruit, suggesting that its progenitors originated from the SSF group. To further explore the tetraploid origin of *A*. *valvata*, we analyzed genome sequences of 16 *Actinidia* species with available published genomes, including five species (*A*. *longicarpa*, *A*. *macrosperma*, *A*. *polygama*, *A*. *reticulata*, and *A*. *rufa*.) that we recently sequenced^[^ ^52^ ^]^ and others from previous studies.^[^ ^22^, ^46^, ^53^, ^54^ ^]^ The phylogeny of *Actinidia* species based on 356 syntenic single‐copy orthologs was consistent with previous reports,^[^ ^51^, ^52^ ^]^ and grouped subgenome A of *A*. *valvata* with *A*. *macrosperma* and subgenome B with *A*. *polygama* (Figure 1d,e; Figure S5a, Supporting Information), corroborating the allotetraploid origin of *A*. *valvata* within the SSF group. Moreover, we assembled a 154 559 bp chloroplast genome for *A*. *valvata*. Phylogenetic analysis based on whole chloroplast genome alignments suggested a close relationship of *A*. *valvata* with *A*. *polygama*, indicating that *A*. *macrosperma* is likely the paternal origin of *A*. *valvata* (Figure S5b, Supporting Information). Estimation of divergence times among *Actinidia* species revealed that subgenome A diverged from *A*. *macrosperma* ≈1.7 million years ago (mya), while subgenome B diverged from its last common ancestor with *A*. *polygama* ≈3.1 mya (Figure 1d). Therefore, the tetraploidization event leading to the emergence of *A*. *valvata* likely occurred ≈1.7 mya.

### 2.3 Unbiased Fractionation and Subgenome Dominance

**Figure 2:**
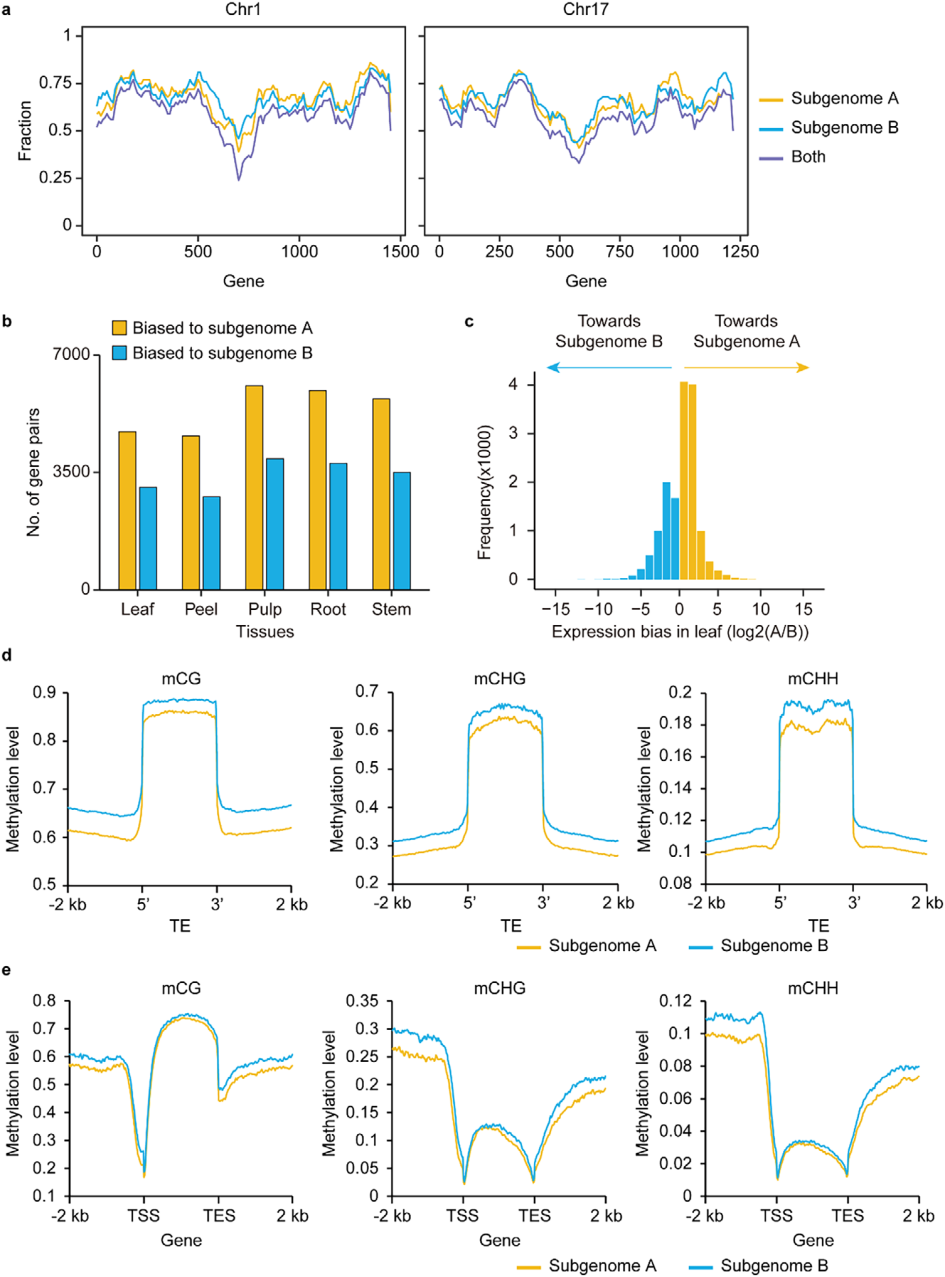
Subgenome dominance in *A*. *valvata*. a) Fractionation patterns in pairs of homoeologous regions in *A*. *valvata*. The average gene retention rates are presented within a 100‐gene window for each subgenome. Retention of genes in subgenomes A,B, and both subgenomes are depicted with different colored lines. Chromosomes (Chr) 1 and 17 are shown here, with other chromosomes displayed in Figure S6 (Supporting Information). b) Number of differentially expressed homoeologs in each subgenome of *A*. *valvata* across five different tissues. c) Distribution of homoeolog expression in leaf samples. Data for other tissues are displayed in Figure S8 (Supporting Information). Only genes with an adjusted *p* value <0.01 are shown. d) Methylation levels of CG, CHG, and CHH in TE bodies, as well as 2 kb flanking regions, in both subgenomes. e) Methylation levels of CG, CHG, and CHH in gene bodies, as well as 2 kb flanking regions, in both subgenomes.

Following polyploidization, duplicated genes may undergo sub‐functionalization or neo‐functionalization to be preserved or to be reduced to a single copy through genome fractionation. This process can involve either biased^[^ ^17^, ^18^, ^55^, ^56^ ^]^ or unbiased^[^ ^57^, ^58^, ^59^, ^60^ ^]^ gene loss toward a single subgenome. In this study, we explored genome fractionation of *A*. *valvata* by analyzing homoeologous gene pairs and singletons within its two subgenomes, which also shared syntenic orthologs with *A*. *chinensis*. Our findings revealed that the subgenomes of *A*. *valvata* maintained a comparable number of genes across chromosomes, suggesting unbiased fractionation after polyploidization (**Figure** **2**a; Figure S6, Supporting Information). To examine the expression of *A*. *valvata* homoeologs, we generated RNA‐Seq data for five tissues, leaf, root, stem, peel, and pulp, each with three biological replicates. PCA analysis showed that samples from the same tissues clustered together (Figure S7a, Supporting Information). We identified a total of 78 583 genes expressed (transcripts per million (TPM) > 0.1) in at least one tissue, with 41 284 from subgenome A and 36 705 from subgenome B (Figure S7b, Supporting Information). Among the expressed homoeologous gene pairs (*n* = 25 376), 69.9% (*n* = 17 747) exhibited expression bias toward one subgenome, predominantly favoring subgenome A across all five tissues (Figure 2b,c; Figure S8, Supporting Information). Furthermore, we compared expression levels of single‐copy genes for which the homoeolog was lost in one subgenome. The expression levels of single‐copy genes in subgenome A (*n* = 18 112) were significantly higher (*p* = 0.00023, Wilcoxon test) than those in subgenome B (*n* = 17 317), corroborating the dominance of subgenome A over subgenome B (Figure S9, Supporting Information).

Gene expression dominance is frequently associated with differential TE load between subgenomes.^[^ ^61^ ^]^ In *A*. *valvata*, TEs constituted 49.19% of the total genomic content in subgenome B, compared to 45.50% in subgenome A (Table S3, Supporting Information). The increased abundance of TEs in subgenome B resulted in their closer proximity to genes, with a median distance of ≈406 bp, which was significantly shorter (*p* = 4.106e‐14, Wilcoxon test) than the distance of 429 bp in subgenome A. Genomic regions harboring TEs are often highly methylated in eukaryotic organisms.^[^ ^62^ ^]^ To elucidate variations in DNA methylation between the subgenomes of *A*. *valvata*, we profiled the DNA methylome in leaves. The global average methylation levels of mCG, mCHG, and mCHH were 64.3%, 24.9%, and 9.0% for subgenome A, respectively, and 69.3%, 29.0%, and 9.8% for subgenome B. Concordantly, DNA methylation levels of TEs and their 2 kb flanking regions were significantly higher in subgenome B compared to subgenome A (Figure 2d). In contrast, DNA methylation within gene bodies was comparable between the two subgenomes; however, methylation levels in the 2 kb upstream and downstream regions were markedly higher in subgenome B than in subgenome A (Figure 2e). This discrepancy is likely due to the spread of DNA methylation from adjacent TEs toward gene regions. While the underlying reasons for the elevated DNA methylation levels of TEs in subgenome B remain unclear, such differential DNA methylation may influence promoter activity, potentially contributing to the observed subgenome expression dominance in the tetraploid *A*. *valvata*.

### 2.4 Dominant Subgenome Exhibits Increased Chromatin Accessibility

**Figure 3:**
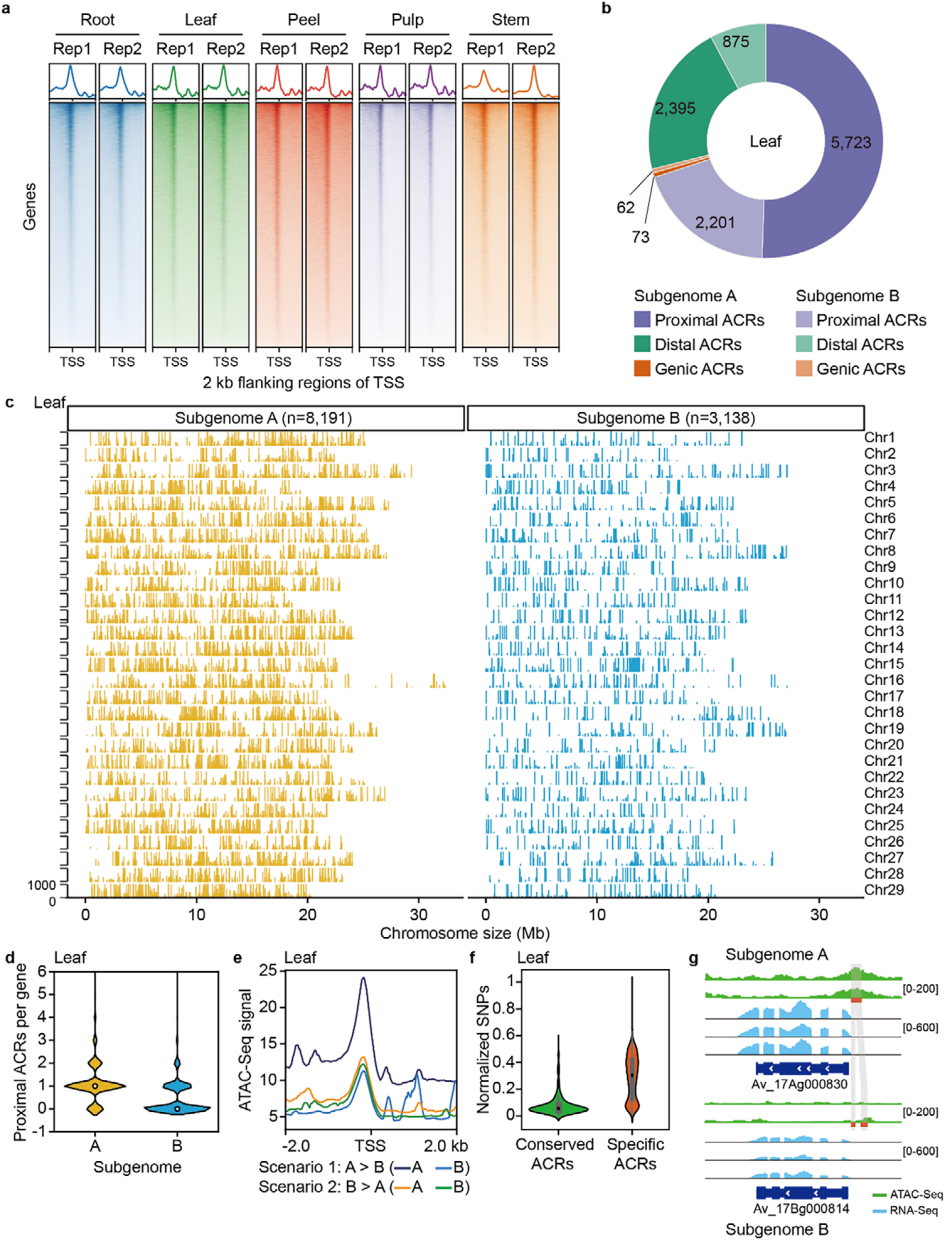
Dominant subgenome exhibits greater chromatin accessibility. a) Enrichment of ATAC‐Seq reads around the transcriptional start sites (TSS) in *A*. *valvata* across five different tissues. b) Distribution of accessible chromatin regions (ACRs) in the *A*. *valvata* genome. ACRs within 5 kb flanking regions of genes are defined as proximal, while those beyond 5 kb are defined as distal. The distribution in leaf sample is displayed here; other tissues are shown in Figure S10, Supporting Information. c) Distribution of ACRs across the two subgenomes in leaf tissue. d) Distribution of proximal ACRs in the two subgenomes. Statistical significance was analyzed using Wilcoxon test. e) Accessibility of genomic regions containing homoeologs with expression bias. Two scenarios are depicted: one where the subgenome A locus is more highly expressed than the subgenome B locus (A>B), and the other where the subgenome B locus is more highly expressed than the subgenome A locus (B>A). f) Normalized SNP numbers between the two subgenomes in conserved or specific ACRs. g) An example illustrating a 126 bp insertion leading to the loss of the proximal ACR and its association with gene expression variation between the subgenomes.

Chromatin accessibility is a key factor determining gene expression and a significant component of epigenetic regulation marked by DNA methylation.^[^ ^63^, ^64^ ^]^ To investigate chromatin accessibility across the two subgenomes of *A. valvata*, we performed ATAC‐Seq on various tissues (**Figure** **3**a). The biological replicates showed high reproducibility (Figure S10a, Supporting Information), enabling the identification of 11 329, 21 402, 38 331, 25 949, and 17 918 highly confident accessible chromatin regions (ACRs) in leaf, peel, pulp, root, and stem tissues, respectively (Figure 3b; Figure S10b, Supporting Information). Among these ACRs, 5389 were conserved across all five tissues, while the remaining were dynamic (Figure S10c, Supporting Information). We grouped 1000 tissue‐specific ACRs ranked by significance in each tissue, and found that each cluster closely matched the functional roles of the respective tissues. For example, peel and pulp‐specific ACRs overlapped with genes enriched with those involved in “specification of floral organ identity,” while genes associated with stem‐specific ACRs were related to xylan biogenesis (Figure S10d,e, Supporting Information).

We classified ACRs based on their proximity to the nearest genes and found that 7924–25 953 ACRs (67.7–71.7%) were located within 5 kb upstream of the transcription start sites (TSS), 135–373 (1.0–1.2%) overlapped with gene bodies, and 3270–12 005 (27.3–31.3%) were in distal regions (>5 kb upstream of TSS) (Figure 3b; Figure S10b, Supporting Information). Consistent with the observed expression dominance and DNA methylation profiles, subgenome A harbored a higher number of ACRs (8191–25 868) compared to subgenome B (3138–12 463) in each tissue examined (Figure 3c; Figure S10b, Supporting Information). After normalizing the number of ACRs by gene number in each subgenome, subgenome A harbored a significantly higher (*p* < 2.2e‐16, Wilcoxon test) number of proximal ACRs per gene compared to subgenome B (Figure 3d; Figure S11a, Supporting Information). Moreover, analysis of ACRs associated with transcriptional divergence of homoeologs showing expression bias revealed a markedly higher level of chromatin accessibility in the upstream regions of subgenome A genes in homoeologous pairs where subgenome A genes were more highly expressed than subgenome B genes (Figure 3e; Figure S11b, Supporting Information). These results suggest that ACRs may serve as major contributors to the observed subgenome dominance in *A. valvata*.

### 2.5 ACR Divergence is Associated with Sequence Variation and Predates Tetraploidization

Given the remarkable differences in ACR numbers between subgenomes A and B, we investigated whether these differences originated in the progenitors of the subgenomes or emerged after tetraploidization. We categorized ACRs into conserved ACRs, which were present in both subgenomes at the same locus, and specific ACRs, which appeared only in one subgenome. Comparing sequence identity between syntenic homoeologous sequences of ACRs in both subgenomes showed that genome regions harboring specific ACRs were significantly more divergent between the two subgenomes than those harboring conserved ACRs. This suggests that genome evolution contributed to the emergence of specific ACRs (Figure 3f; Figure S11c, Supporting Information). To determine whether these DNA variations were inherited or occurred after polyploidization, we examined the syntenic regions of ACRs in both *A*. *polygama* and *A*. *macrosperma*. Among 50 722 SNPs/InDels found in subgenome‐specific ACR regions, 39 203 (77.3%) had the same genotype between subgenome A and *A*. *macrosperma*, or between subgenome B and *A*. *polygama*. A notable example was the locus *Av_17Ag000830* (subgenome A) and its homoeolog *Av_17Bg000814* (subgenome B), where a 126 bp DNA insertion resulted in the loss of the proximal ACR in *Av_17Bg000814*, likely contributing to its reduced gene expression, while the ACR persisted in subgenome A (Figure 3g). This insertion variation was also observed in the comparison of *A*. *polygama* and *A*. *macrosperma* genomes. Overall, our data indicate that ACR divergence in the subgenomes of *A*. *valvata* was predominantly driven by the genome evolution of ancestral species and predated polyploidization.

### 2.6 Subgenome A is Transcriptionally more Responsive to Waterlogging Stress

**Figure 4:**
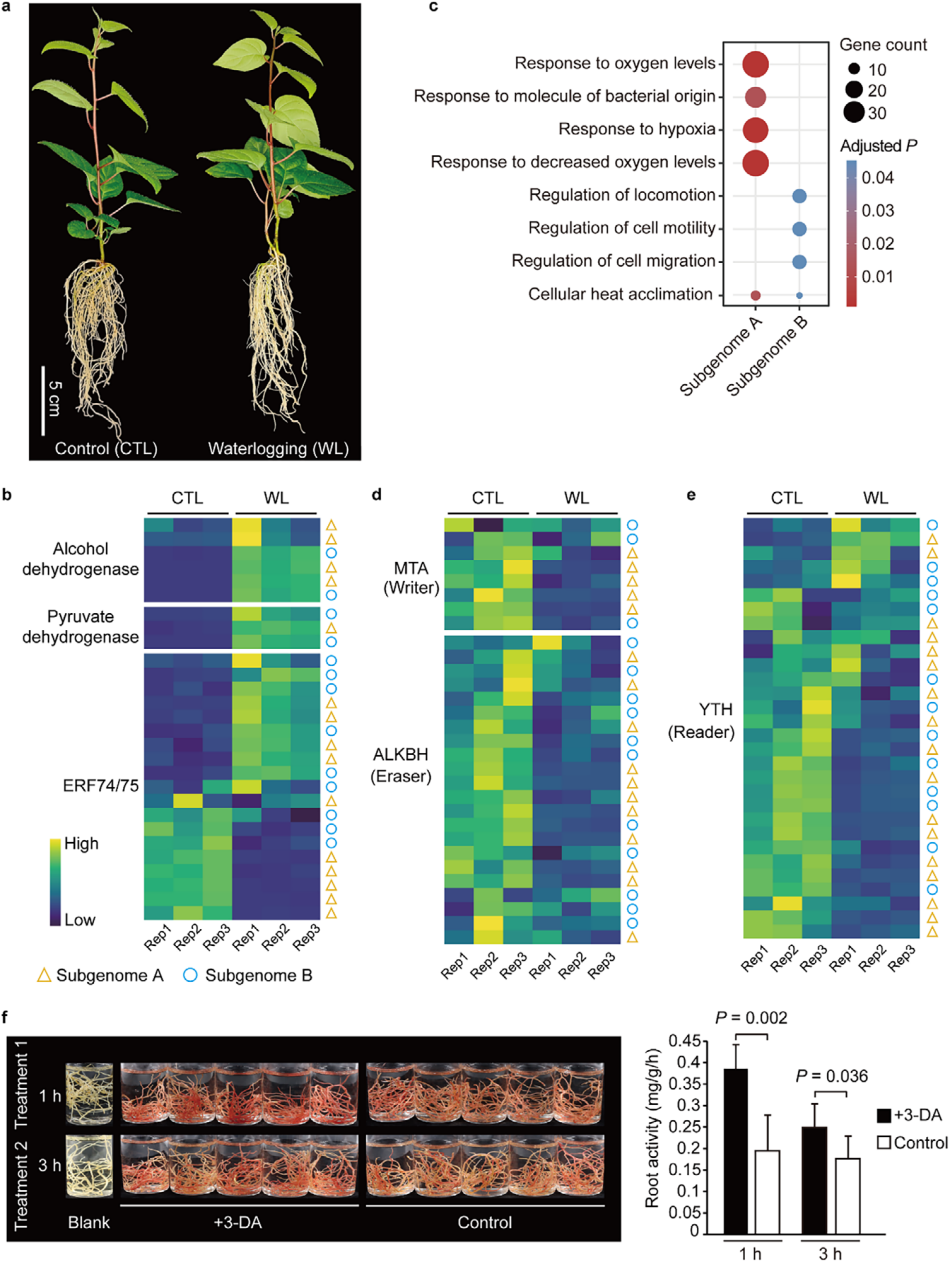
Morphological and transcriptional responses of *A*. *valvata* roots to waterlogging treatment. a) *A*. *valvata* plants subjected to waterlogging stress (WL) and control (CTL) conditions. b) Expression of genes involved in anaerobic fermentation under WL and CTL conditions. Genes from subgenome A and subgenome B are marked with red triangle and blue circle, respectively. c) Biological processes enriched in genes responsive to waterlogging stress in the two subgenomes. d,e) Expression of genes involved in RNA m^6^A methylation. MTA: mRNA adenosine methylase; ALKBH: alkB homolog; YTH: YT521‐B homology. f) Roots of *A*. *valvata* were immersed in either water (control) or water containing 3‐DA for 1 or 3 h to assess their activity. Statistical significance was calculated using two‐tailed Student's *t*‐test. Data are presented as mean ± SD.

The kiwifruit industry faces significant threats from waterlogging stress, which adversely impacts plant growth and yield. Cultivated kiwifruits are sensitive to waterlogging, whereas *A*. *valvata* exhibits strong tolerance (Figure S12, Supporting Information). Previous screening of kiwifruit germplasm revealed that *A*. *macrosperma*, the closest relative of the subgenome A of *A*. *valvata*, also showed excellent waterlogging tolerance.^[^ ^34^, ^65^ ^]^ This suggests that the origin of waterlogging tolerance in *A*. *valvata* may be linked to its subgenome A. To gain further insights into the molecular mechanisms underlying waterlogging tolerance in *A*. *valvata*, we performed RNA‐Seq analysis on *A*. *valvata* soil roots subjected to waterlogging for 3 days, at which stress symptoms started to appear in susceptible cultivars but remained less obvious in *A*. *valvata* (**Figure** **4**a). We identified 9814 downregulated genes and 7657 upregulated genes under waterlogging treatment compared to control. Among these differentially expressed genes (DEGs), those encoding alcohol dehydrogenase, pyruvate dehydrogenases, and ERF 74/75 transcription factors, key markers of plant metabolic shifts under waterlogging stress, were significantly upregulated (Figure 4b). Gene Ontology (GO) analysis of DEGs revealed a significant enrichment of upregulated genes in biological processes related to hypoxia or oxygen level responses (Figure 4c). This enrichment was particularly notable in subgenome A (Figure 4c), agreeing with the fact that subgenome A (*n* = 9382; 20%) had more DEGs compared to subgenome B (*n* = 8089; 19%) under waterlogging treatment. These findings imply that subgenome A plays a more important role in mediating waterlogging responses in *A*. *valvata*.

### 2.7 RNA m^6^A Modification Regulates Waterlogging Response in *A*. *valvata*

Our gene expression analysis revealed significant transcriptional perturbations in genes associated with RNA m^6^A modification under waterlogging stress. Specifically, we observed a global downregulation of both m^6^A writers and erasers, while most m^6^A readers exhibited differential expression patterns, with a predominant trend toward downregulation (Figure 4d,e). RNA m^6^A modification is known to play a critical role in stress responses, including hypoxia, in both animals and plants.^[^ ^66^, ^67^, ^68^, ^69^ ^]^ To investigate whether RNA m^6^A modification regulates waterlogging responses, we treated the roots with 3‐deazaneplanocin A (3‐DA), an inhibitor of m^6^A writers commonly used in plants.^[^ ^70^, ^71^ ^]^ Although root activity of *A*. *valvata* decreased over the duration of waterlogging treatment, the addition of 3‐DA significantly increased root activity during both 1‐ and 3‐h waterlogging treatments compared to the control (Figure 4f), confirming the involvement of RNA m^6^A modification in the waterlogging response of *A*. *valvata*.

**Figure 5:**
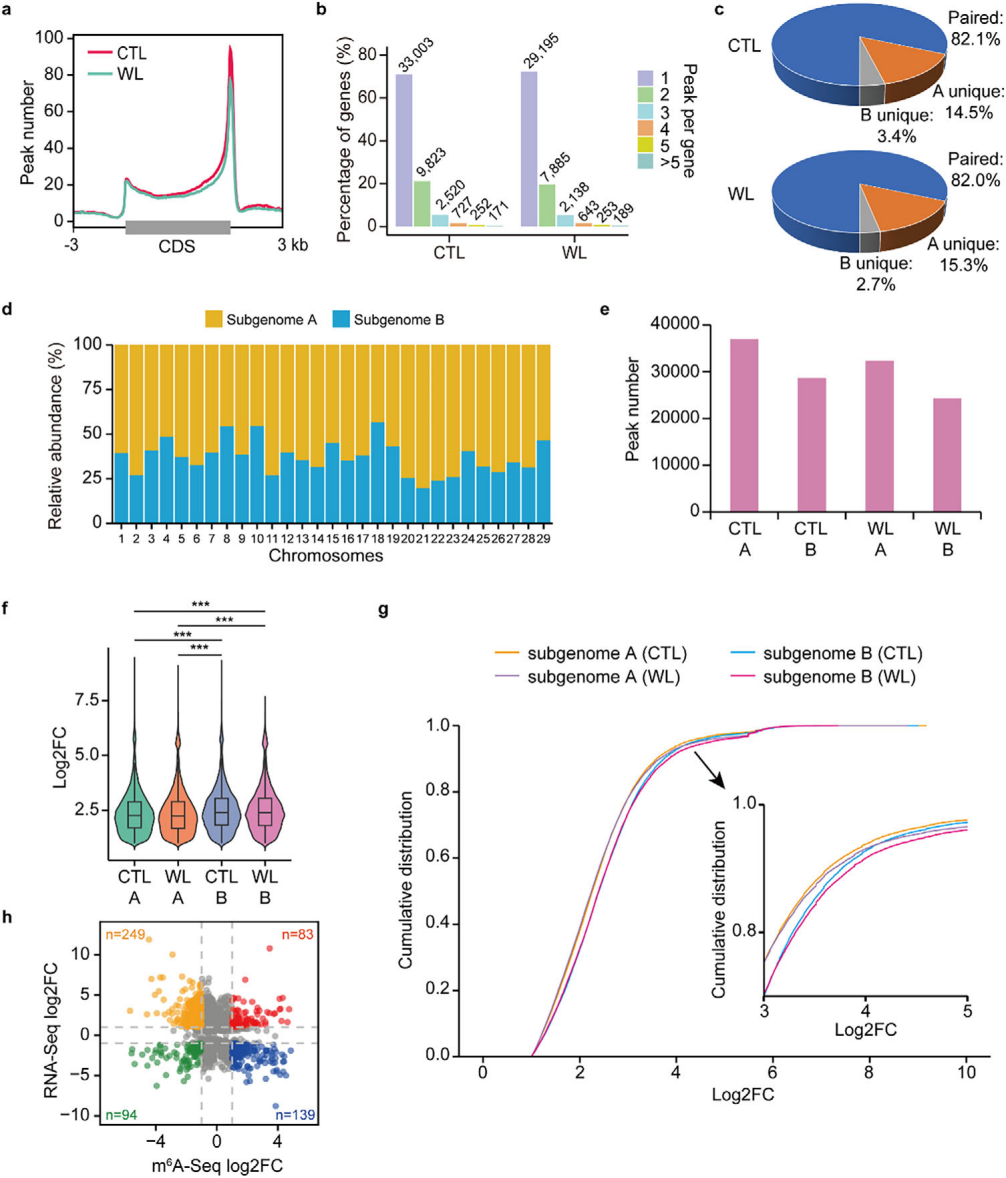
RNA m^6^A modification in *A*. *valvata*. a) Enrichment of RNA m^6^A modification in 3’‐UTRs in control (CTL) and waterlogged (WL) roots. b) Summary of m^6^A peaks associated with genes in the *A. valvata* genome. c) Summary of m^6^A peaks between homoeologs in the two subgenomes. d) Distribution of m^6^A peaks in different chromosomes. e) Number of m^6^A peaks identified in CTL and WL roots. f) Distribution of methylation levels, measured by log_2_ fold change (FC) of IP to Input ratio in CTL and WL roots. Statistical significance was analyzed using Wilcoxon test (*** denotes *p <* 0.001). g) Cumulative distribution of methylation levels in CTL and WL samples. h) Intersection of genes showing significant changes in gene expression and m^6^A modifications under WL.

To further explore the mechanisms of m^6^A modification, we performed m^6^A‐seq on soil root samples under waterlogged and control conditions (Figure S13, Supporting Information). We identified 65 586 and 56 579 confident m^6^A peaks (IP/input ≥ 2, *p* value < 0.05) associated with 46 496 and 40 303 genes in the control and waterlogged samples, respectively. These m^6^A peaks were predominantly located in 3’‐UTRs (**Figure** **5**a), with the majority of genes containing a single m^6^A peak (Figure 5b). Among the genes with m^6^A modifications, ≈82% were homoeologous pairs present in both subgenomes, while ≈15% were specific to subgenome A, compared to only ≈3% for subgenome B (Figure 5c,d). This differential distribution of m^6^A modifications suggests a potential mechanism of subgenome dominance at the level of post‐transcriptional regulation.

We compared m^6^A peaks between waterlogged and control samples and found a 15.9% decrease in peak numbers under waterlogging for both subgenomes (Figure 5e). Furthermore, subgenome A exhibited a significantly but slightly lower global RNA m^6^A methylation level, measured by log_2_(IP/input), compared to subgenome B under both conditions (Figure 5f,g). Among m^6^A peaks detected under both control and waterlogging conditions, 1126 in subgenome A and 641 in subgenome B exhibited differential methylation levels (Figure 5g). Integrative analysis of RNA‐Seq and m^6^A‐Seq data showed that m^6^A modification was associated with both the promotion and repression of mRNA transcription in *A*. *valvata*, with its repressive effects being more significant (Figure 5h), aligning with recent reports in other plants.^[^ ^72^ ^]^ Together, these findings indicate that both m^6^A methylation abundance and distribution contribute to the transcriptional regulation of waterlogging‐responsive genes, likely in a subgenome‐biased manner.

### 2.8 Waterlogging‐Responsive Genes are Distinctively Regulated by m^6^A Modifications

**Figure 6:**
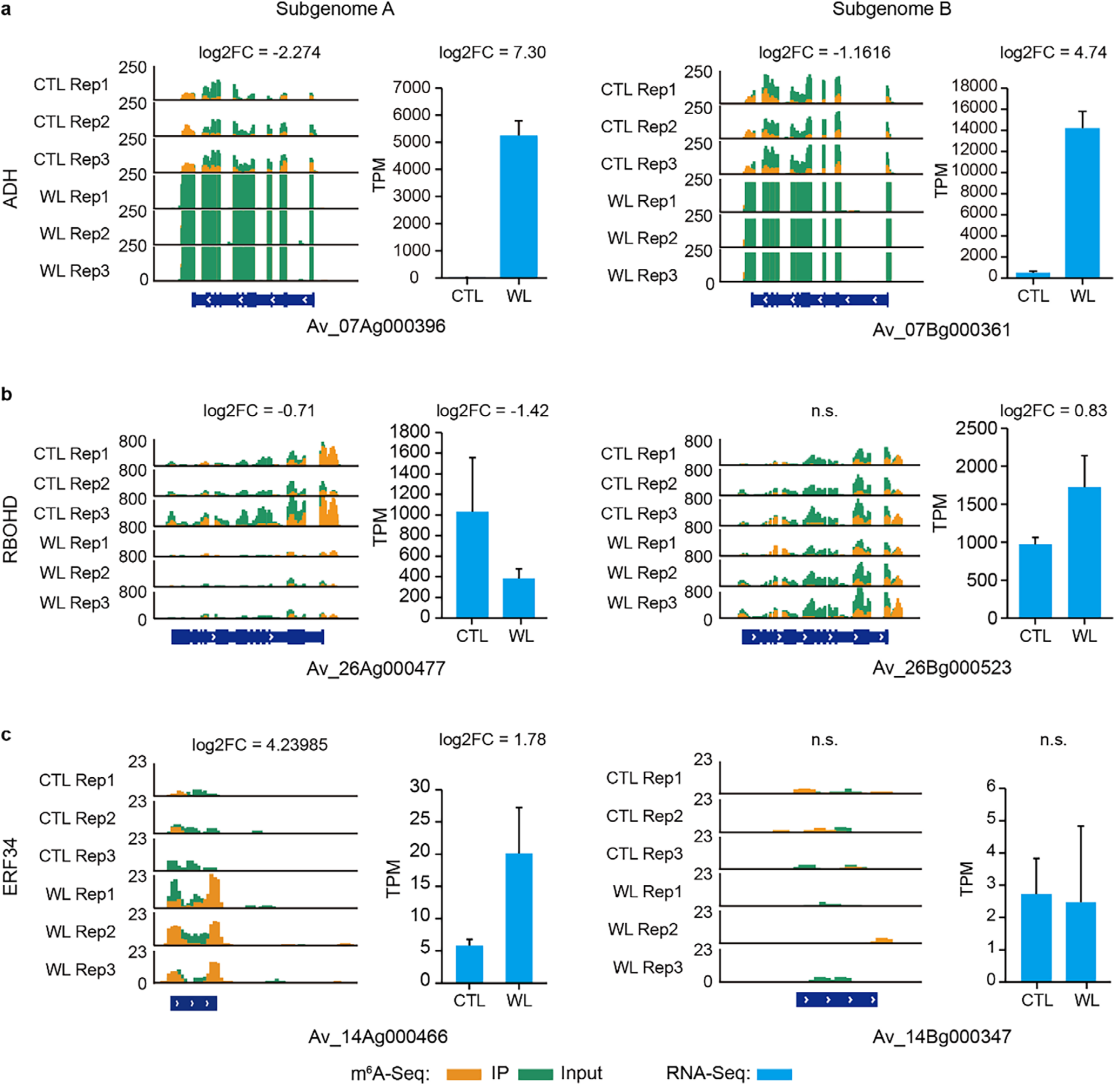
Profiles of expression and m^6^A modifications in selected genes. Genes presented here encode an alcohol dehydrogenase (ADH) a), the respiratory burst oxidase homolog D (RBOHD) b), and the ERF34 transcription factor c). For each gene, left panel shows m^6^A modifications in the control (CTL) and waterlogged (WL) soil roots, while right panel displays its gene expression levels presented as mean ± SD. TPM, Transcripts Per Million.

Among genes exhibiting changes at both transcription and m^6^A modification levels under waterlogging conditions, we found several of them were associated with waterlogging responses. For example, waterlogging significantly induced the expression of a homoeologous pair of alcohol dehydrogenase (ADH) genes, *Av_07Ag000396* and *Av_07Bg000361*, with a concomitant reduction in m^6^A modification of their mRNAs (**Figure** **6**a). Waterlogging‐triggered ROS production is dependent on the ROS‐generating RESPIRATORY BURST OXIDASE HOMOLOG D (RBOHD).^[^ ^73^ ^]^ The ability to control ROS production to mitigate cell death under waterlogging conditions is an important mechanism for waterlogging tolerance. We found that the expression of RBOHD gene *Av_26Ag000477* from subgenome A was suppressed after 3 days of waterlogging, whereas its homoeolog from subgenome B remained activated. Correspondingly, the m^6^A modification of *Av_26Ag000477* was significantly reduced, while no change was observed for its subgenome B homoeolog (Figure 6b). Furthermore, subgenome A homoeologs of some ERF transcription factors, which are known for mediating various stress responses, such as ERF34^[^ ^74^ ^]^ (*Av_14Ag000466*), displayed upregulated expression and m^6^A modification changes, while their subgenome B homoeologs exhibited no significant expression and m^6^A modification change (Figure 6c). Additionally, we quantified the expression of ADH and RBOHD genes in 3‐DA‐treated roots using RT‐qPCR (Figure 4f) and observed expression patterns consistent with transcriptome data of waterlogged soil roots (Figure 6; Figure S14, Supporting Information). These results demonstrate that RNA m^6^A modification is modulated by waterlogging stress, with varying tolerance associated with distinct patterns of m^6^A methylation and transcriptional regulation in key waterlogging‐responsive genes.

## 3 Discussion

Polyploidy in plants is pervasive and has been identified as a critical driving force of evolution and speciation.^[^ ^1^ ^]^ Polyploid species, derived from genetically distinct diploid parents, exhibit increased genetic variation, enhanced genomic complexity, and greater environmental adaptability.^[^ ^75^, ^76^ ^]^ Consequently, creating polyploid hybrids represents a potent approach for generating novel genetic diversity that is beneficial for breeding programs.^[^ ^77^ ^]^ Kiwifruits exhibit complex ploidy variations in nature.^[^ ^78^ ^]^ Many kiwifruit cultivars are polyploids, either occurring naturally or developed by breeders. A comprehensive understanding of genetic variation related to ploidy levels is critical for elucidating how polyploidy contributes to the diversity of kiwifruit germplasm. Our assembled genome of tetraploid *A*. *valvata*, which produces viable seeds with normal germination, spans 1.36 Gb of genomic sequences, with a total of 58 chromosomes covering ≈98.4% of the *A*. *valvata* genome. This reference genome assembly significantly enriches the genomic resources for *Actinidia* species, facilitating functional genomic studies, evolutionary research, and gene mining for the improvement of related *Actinidia* species. Consequently, this assembly meets the critical needs of the kiwifruit industry by providing a robust genomic framework for future breeding and research.

Comparative genomic analysis revealed that the tetraploidization event in *A*. *valvata* occurred ≈1.7 mya, predating the polyploidy events observed in common crops such as cotton (1.0–1.6 mya)^[^ ^79^ ^]^ and wheat (0.5 mya).^[^ ^80^ ^]^ Our analysis demonstrated closer phylogenetic relationships between subgenome A of *A*. *valvata* and *A*. *macrosperma*, and between subgenome B and *A*. *polygama*, suggesting these two species as the putative ancestors of *A*. *valvata*. Additionally, our results indicated that 47.28% of the assembled *A*. *valvata* genome is annotated as LTR‐RTs, predominantly consisting of *Copia* and *Gypsy* elements. Notably, the estimated insertion times suggest an ancient expansion of *Copia* LTR‐RTs (≈5 mya), which contrasts with many other plants and accounts for their prevalence over *Gypsy* LTR‐RTs (Figure 1c).

Hybridization or allopolyploidization can result in “genome shock,” often manifested as subgenome dominance.^[^ ^81^ ^]^ The dominant subgenome typically exhibits higher overall gene expression due to biased genetic and epigenetic modifications.^[^ ^82^ ^]^ Our study found that *A*. *valvata* also exhibits subgenome dominance, with significantly higher expression levels of genes in subgenome A compared to subgenome B. In allopolyploids, TEs are implicated in subgenome dominance due to their ability to suppress the expression of neighboring genes. In the case of *A*. *valvata*, our analysis revealed that TE content constitutes 49.19% of subgenome B, compared to 45.50% in subgenome A (Table S3, Supporting Information). Considering that genomic regions containing TEs are often highly methylated in eukaryotic organisms,^[^ ^62^ ^]^ we performed DNA methylation analysis. Our results indicated that subgenome A exhibits a lower global average methylation level compared to subgenome B, consistent with its reduced TE content. These results collectively support the dominance of subgenome A over subgenome B in *A*. *valvata*.

The gain or loss of DNA sequences flanking ACRs can significantly impact the balance of expression levels and/or tissue specificity of duplicated or homoeologous genes, as evidenced in soybean^[^ ^83^ ^]^ and strawberry.^[^ ^84^ ^]^ In this study, we observed that, similar to expression dominance and DNA methylation profiles, subgenome A of *A*. *valvata* harbors a greater number of ACRs and exhibits fewer mutations within these regions compared to subgenome B (Figure 3c). Notably, subgenome A demonstrated a significantly higher number of proximal ACRs per gene relative to subgenome B (Figure 3c; Figure S11a, Supporting Information). Furthermore, for homoeologous genes displaying expression bias, the upstream regions of genes in subgenome A showed enhanced chromatin accessibility compared to their counterparts in subgenome B. These results implicate that the gene expression dominance of subgenome A is mediated by the abundance and accessibility of ACRs associated with the respective genes.

Recently, RNA m^6^A methylation, a critical conserved epigenetic modification, has been recognized as a key regulator of various biological processes, including development, fruit ripening, and biotic/abiotic stress responses.^[^ ^44^ ^]^ Waterlogging‐induced damage primarily stems from oxygen deficiency, which leads to hypoxic or anoxic conditions.^[^ ^85^ ^]^ In animals, m^6^A modifications play a pivotal role in hypoxic stress response, with significant upregulation of m^6^A levels and METTL3‐mediated m^6^A modification being identified as essential mechanisms in endothelial cells and mouse retinas.^[^ ^66^ ^]^ In this study, we observed a global reduction in m^6^A peaks in *A*. *valvata* after three days of waterlogging, accompanied by altered expression of the majority of m^6^A‐related and hypoxia‐responsive genes. Inhibition of m^6^A writers led to increased root activity under waterlogging conditions, suggesting that m^6^A modification is involved in the waterlogging stress response in *A*. *valvata*. Although the exact mechanisms remain to be elucidated, our data indicate that both increases and decreases in m^6^A levels, particularly on waterlogging‐responsive genes, may influence resistance to waterlogging stress. Similarly, inhibition of either m^6^A writers or erasers has been shown to suppress fruit expansion in tomato.^[^ ^86^ ^]^ Given that m^6^A deposition on coding sequences or 3’UTR regions exert opposite effects on mRNA abundance,^[^ ^87^, ^88^ ^]^ m^6^A likely functions in distinct ways across different genes to regulate biological processes. For instance, the observed decrease in m^6^A modification on alcohol dehydrogenase (ADH), a key enzyme in anaerobic metabolism that is rapidly induced in kiwifruit roots to enhance fermentation under hypoxic conditions,^[^ ^89^ ^]^ may enhance its transcript stability, thereby contributing to waterlogging tolerance in *A*. *valvata*.

## 4 Conclusion

In conclusion, our findings shed light on the intricate mechanisms underlying subgenome dominance in *A*. *valvata*, and provide a robust foundation for genetic design and precision breeding to enhance the resilience of kiwifruits in the future.

## 5 Experimental Section

### Plant Material

The *A. valvata* plant used in this study was grown in the germplasm of Zhejiang A&F University. Voucher specimens (ZAFU 25001) of *A. valvata* were deposited at the Herbarium of Zhejiang Agricultural and Forestry University (ZJFC).

### DNA Sequencing

Seedlings of *A. valvata* were grown in a greenhouse at Zhejiang A&F University under a 16/8 h day/night cycle at 25 °C. Young, fresh leaves were collected from the plants, and genomic DNA was isolated using the CTAB method. The DNA was used to construct PacBio SMRTbell libraries following the standard protocol (PacBio, USA). The constructed libraries were sequenced on the PacBio Sequel II platform. The same genomic DNA was also used to construct a paired‐end library with insert sizes of 300–500 bp using the MGIEasy PCR‐Free DNA Library Prep Kit (BGI, China), following the manufacturer's instructions. This library was sequenced on the BGI DNBSEQ‐T7 platform. Additionally, a Hi‐C library was constructed using young leaves following the protocol of Hi‐C Library Prep Kit for Illumina and sequenced on the NovaSeq 6000 platform. Raw reads were filtered to remove adaptors and duplicates before downstream analyses.

To facilitate gene prediction, RNA‐Seq was performed using pooled tissues. Total RNA was extracted using the TRIzol method and quantified with a NanoDrop ND‐2000 spectrophotometer (NanoDrop Technologies). mRNA, purified from total RNA with a RIN score ≥8 (Bioanalyzer 2100, Agilent Technologies), was used to construct non‐stranded RNA‐Seq libraries with the NEBNext Ultra II RNA Library Prep Kit for Illumina (NEB), following the manufacturer's instructions. The RNA‐Seq libraries were sequenced on an Illumina NovaSeq 6000 platform under the 2 × 150 bp mode.

### Genome Assembly and Annotation

PacBio HiFi reads were used for *de novo* assembly with Hifiasm (v0.19.5‐r590) using default parameters.^[^ ^72^ ^]^ Short reads were aligned to the assembled contigs using BWA (v0.7.15) (‐men mode)^[^ ^90^ ^]^ and error correction was then performed using Pilon (v1.22) with default parameters.^[^ ^91^ ^]^ Redundant contigs in the polished assembly were removed using Purge Haplotigs (parameters: default).^[^ ^92^ ^]^ Hi‐C data were used to produce pseudochromosomes with the 3D‐DNA program (parameters: default).^[^ ^93^ ^]^ Juicebox^[^ ^94^ ^]^ was utilized to visualize the Hi‐C contact matrix, and manual corrections were performed based on neighboring interactions and genome synteny. A species‐specific TE library was constructed using EDTA software (v2.0.0) (parameters: default),^[^ ^95^ ^]^ which was then used to generate a repeat masked genome for gene prediction, achieved using BRAKER3 (parameters: default).^[^ ^96^ ^]^ The quality of the genome assembly and the predicted gene set was assessed by examining the coverage of highly conserved genes using BUSCO (parameters: ‐l embryophyta_odb10 ‐m genome/protein).^[^ ^48^ ^]^ Functional annotation of genes was performed by searching protein sequences against public databases, including NR, InterPro, KEGG, and eggNOG.

### Subgenome Phasing

SubPhaser^[^ ^45^ ^]^ was used to cluster chromosomes into two groups based on the subgenome‐specific *k*‐mers (k = 15). Unsupervised hierarchical clustering and PCA of the differential 15‐mers validated that the genome could be successfully phased into two subgenomes based on clearly distinct patterns of both differential *k*‐mers and homoeologous chromosomes.

### Phylogenetic and Comparative Genomic Analysis

Orthogroups of the representative species were identified using OrthoFinder (v2.5.5) (parameters: default).^[^ ^97^ ^]^ Low‐copy genes from these orthogroups were extracted and aligned separately using MAFFT (linsi mode).^[^ ^98^ ^]^ The species phylogeny was examined using two approaches, a maximum likelihood phylogeny constructed from concatenated protein sequence alignments with IQ‐TREE2 (parameters: ‐m TEST)^[^ ^99^ ^]^ and a supertree method implemented in ASTRAL (parameters: default).^[^ ^100^ ^]^ Species divergence times were estimated using the MCMCTree (approximate likelihood method) program in the PAML package,^[^ ^101^ ^]^ with calibration times were retrieved from the TimeTree database.^[^ ^102^ ^]^

### ATAC‐Seq and RNA‐Seq Library Preparation

Leaf, peel, pulp, root, and stem tissues were flash‐frozen in liquid nitrogen immediately after collection. ATAC‐Seq library preparation was performed as described previously.^[^ ^44^ ^]^ Briefly, for each replicate, ≈1 g of frozen tissue was ground into powder and immediately placed in 10 mL of pre‐chilled NIB lysis buffer (9.75 mL HB, 0.25 mL Triton X‐100 mix, 25 µL beta‐mercaptoethanol, and 0.1 g PVP360). The mixture was then shaken at 200 rpm for 15 min. Nuclei were filtered through four layers of Miracloth and centrifuged at 5000 rpm for 20 min at 4 °C. The pellet was resuspended in NIB lysis buffer and centrifuged at 3000 rpm for 10 min at 4 °C. The resulting pellet was resuspended in 1 mL of NBI lysis buffer and centrifuged at 2000 rpm for 5 min at 4 °C. After discarding the supernatant, the isolated nuclei pellet was resuspended in 500 µL of HB. Following quality confirmation of the nuclei, a transposition mix containing TDE buffer and Tn5 enzyme (Illumina, FC‐121‐1031) was added, and the reaction was incubated at 37 °C for 30 min. The entire reaction was used for PCR amplification in a 50 µL reaction using Nextera primers with 11 cycles of amplification. Libraries were cleaned using 0.7× volume of AmpureXP beads (Beckman Coulter) and checked for quality and quantity using a Bioanalyzer (Agilent). All subsequent steps followed the standard ATAC‐seq library protocol.

The same samples were used to extract total RNA with TRIzol Reagent (Thermo Fisher Scientific) following the manufacturer's instructions. For each tissue and replicate, 1.3 µg of total RNA was used for strand‐specific RNA‐Seq library constriction using the Illumina Truseq mRNA Stranded Library Kit (Illumina) following the manufacturer's instructions.

### ATAC‐Seq Data Analysis

ATAC‐Seq raw reads were trimmed to remove adaptors using Trimmomatic (parameters: default),^[^ ^103^ ^]^ and the trimmed reads were mapped to the *A*. *valvata* genome using Bowtie2 (parameters: ‐X 2000).^[^ ^104^ ^]^ Properly mapped alignments were deduplicated and subjected to peak calling using MACS2 (parameters: –keep‐dup all ‐q 0.05 ‐nomodel ‐shift ‐100 ‐extsize 200).^[^ ^105^ ^]^ To visualize the distribution of peaks around TSS, the computeMatrix and plotHeatmap functions in deepTools2 (parameters: ‐b 2000 ‐a 2000 ‐missingDataAsZero –skipZeros)^[^ ^106^ ^]^ were used to generate heatmaps. Peak overlap analysis, peak annotation, differential ACR analysis, motif enrichment analysis and data visualization were performed using the CisDynet platform.^[^ ^107^ ^]^

### M^6^A Sequencing and Analysis

Waterlogging treatment was performed by immersing the *A. valvata* seedling pots in basins with water surface 5 cm above the potting soil. Roots of *A*. *valvata* were harvested from the soil after 3 days under waterlogging and control conditions. Total RNA was extracted using TRIzol reagent (Invitrogen, Carlsbad, CA, USA), followed by purification of poly(A) RNA from the total RNA using oligo(dT)25 Dynabeads (Thermo Fisher Scientific). The purified poly(A) RNA was then fragmented using the Magnesium RNA fragmentation module (NEB). The fragmented RNA was incubated with an m^6^A‐specific antibody (No. 202003, Synaptic Systems, Germany) in IP buffer. The immunoprecipitated (IP) RNA was then reverse‐transcribed into cDNA using SuperScript™ II Reverse Transcriptase (Invitrogen, cat. 1896649, USA). The resulting cDNA was used to synthesize U‐labeled second‐strand DNAs using *E*. *coli* DNA polymerase I (NEB, cat. m0209, USA), RNase H (NEB, cat. m0297, USA), and dUTP solution (Thermo Fisher, cat. R0133, USA). Size selection was performed with AMPureXP beads. The U‐labeled second‐strand DNAs were treated with a heat‐labile UDG enzyme (NEB, cat. m0280, USA), and the ligated products were amplified by PCR. The final cDNA library had an average insert size of 300 ± 50 bp. Sequencing was performed on an Illumina Novaseq 6000 in paired‐end (2×150 bp) mode (LC‐Bio Technology CO., Ltd., Hangzhou, China). Cleaned reads, after the removal of adaptors, were mapped to the reference genome using HISAT2 (parameters: default).^[^ ^108^ ^]^ Mapped reads from IP and Input libraries were analyzed with the R package exomePeak (https://github.com/ZW‐xjtlu/exomePeak) to identify m^6^A peaks. The identified peaks were annotated by intersecting with gene architecture using the R package ChIPseeker (parameters: default).^[^ ^109^ ^]^ FeatureCounts^[^ ^110^ ^]^ (parameters: default) was utilized to quantify expression levels of all mRNAs from Input libraries by calculating transcripts per million (TPM). Differentially expressed mRNAs were identified using DESeq2,^[^ ^111^ ^]^ with selection criteria of a fold change > 2 and a false discovery rate (FDR) < 0.05.

### Whole‐Genome Bisulfite Sequencing and Analysis

100 ng of high‐quality DNA was extracted from *A*. *valvata* leaves and sonicated using the Covaris S220 system. The fragmented DNA (200–300 bp) was then treated with bisulfite using the EZ DNA Methylation‐Gold™ Kit (Zymo Research). The quality of the resulting library was assessed before sequencing on the Illumina NovaSeq 6000 platform in paired‐end (2 × 150 bp) mode. Raw reads were cleaned and subsequently mapped against base transformed reference genome using BatMeth2 (parameters: default).^[^ ^112^ ^]^ Read alignments with mapping quality >20 were preserved to calculate methylation levels in a 1 kb window with or without predefined genomic regions. Methylation levels were calculated using BatMeth2 with default parameters.

### Measurement of Root Activity

Root samples weighing 0.25 g were fully immersed in 10 mL of treatment solution either deionized water (ddH_2_O) or ddH_2_O containing 10 µM 3‐deazaneplanocin A (3‐DA) for durations of 1 and 3 h, respectively. Each treatment group consisted of five individual root samples as biological replicates. Following exposure, root activity was evaluated using a modified version of the method described previously.^[^ ^89^ ^]^ Briefly, 0.25 g of root samples were transferred into a solution containing 5 mL of 0.4% triphenyl tetrazolium chloride (TTC) and an equal volume of 0.1 M phosphate buffer. The mixture was incubated in the dark at 37 °C for 1 h. After incubation, 2 mL of 1 M sulfuric acid was added to terminate the reaction. The roots were then drained, homogenized, and subjected to ethyl acetate extraction to isolate the triphenyl formazan product. The extract was diluted to a final volume of 10 mL, and its absorbance was measured at 485 nm using the blank test as a reference. The blank test was conducted by adding sulfuric acid to the mixture prior to immersing the roots in the solution; all subsequent steps were identical to those of the experimental conditions. The amount of TTC reduction, indicative of root activity, was determined by reference to a standard curve. Root activity was quantified as: root activity = TTC reduction(mg) / (root fresh weight(g)/time(h)).

### Statistical Analysis

Statistical analysis was performed using R. Wilcoxon test and two‐tailed Student's *t*‐test were used for group comparison. Data are presented as the mean ± standard deviation (SD) from at least three independent replicates, and *p* value *<* 0.05 was considered statistically significant (*p < 0.05, **p < 0.01, and ***p < 0.001).

## Conflict of Interest

The authors declare no conflict of interest.

## Author Contributions

X.H. and C.X. contributed equally to this work. X.S. conceived the project. X.H., C.X., X.L., L.L., Y.B., M.G. X.L., X.L., L.H., J.G., M.W., and K.X. collected samples and performed experiments. X.H. and X.S. analyzed the data. X.H. and X.S. wrote the manuscript. Z.F. and X.Y. commented and revised the manuscript.

## Supporting information



Supplemental Tables

Supplemental Figures

## Data Availability

The data that support the findings of this study are openly available in CNGB Sequence Archive (CNSA) of China National GeneBank DataBase (CNGBdb) under the accession number PRJCA027372.
