# Supplementary material for: Subgenome Dominance in Allotetraploid Actinidia valvata Regulates RNA m6A Modification for Waterlogging Tolerance
Source: Adv Sci (Weinh). 2025 Jun 5;12(32):e03974. doi: 10.1002/advs.202503974 (PMC12407272; doi:10.1002/advs.202503974)
Supplement: Supplementary file 2 — Supplemental Figures [file ADVS-12-e03974-s001.pdf]

ADVANCED SCIENCE
Open Access

Supporting Information

for *Adv. Sci.*, DOI 10.1002/advs.202503974

Subgenome Dominance in Allotetraploid *Actinidia valvata* Regulates RNA m^{6}A Modification for Waterlogging Tolerance

Xiaoli Hu, Changbin Xu, Xiaolan Li, Lin Li, Yu Bao, Miaofeng Gu, Xinyi Li, Liuqing Huo, Jinli Gong, Xiaolong Li, Minhui Wang, Kai Xu, Xueren Yin, Zhangjun Fei and Xuepeng Sun**

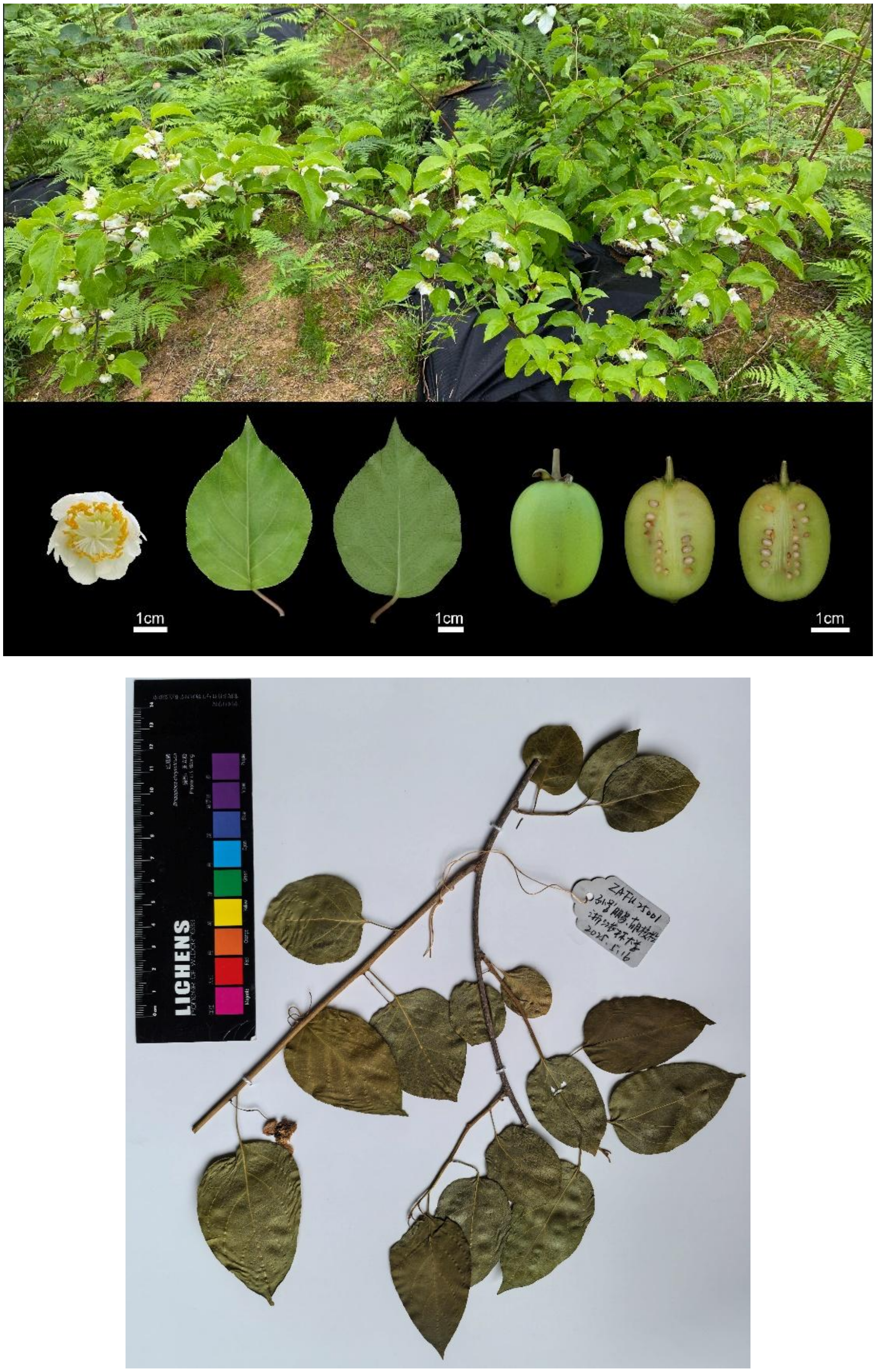

Figure S1. Morphology and voucher specimen of *A. valvata* plants sequenced in this study.

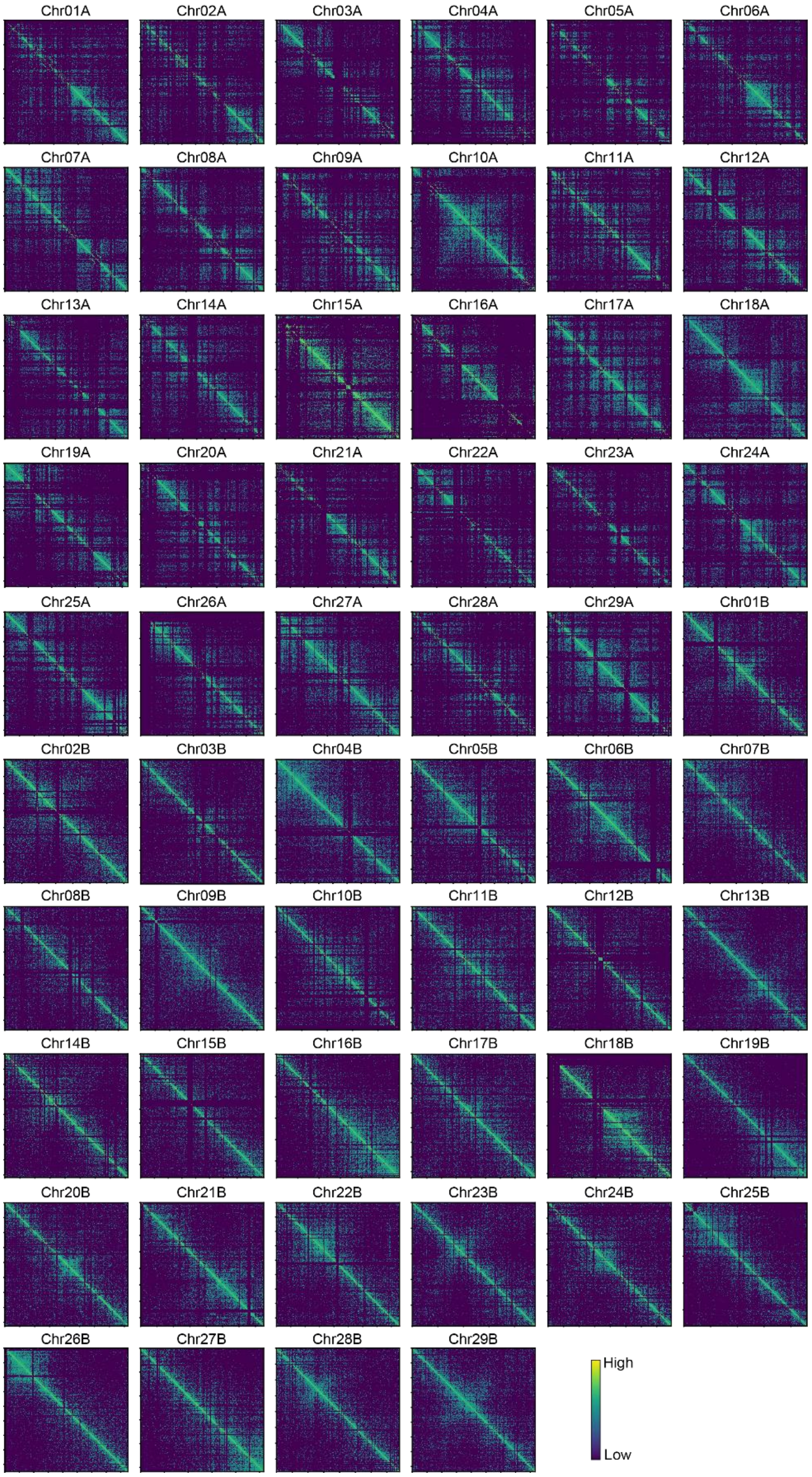

Figure S2. Heatmaps showing chromatin interactions in *A. valvata* based on Hi-C data.

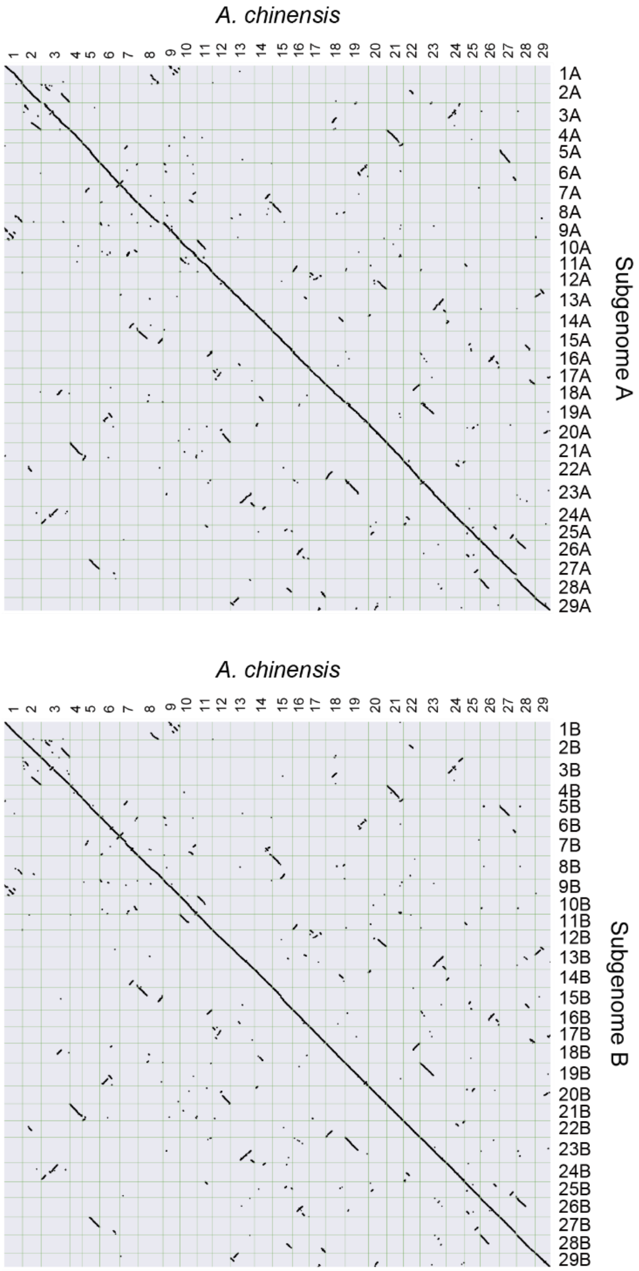

Figure S3. Collinearity between the two subgenomes of *A. valvata* and the genonme of *A. chinensis*. The *A. chinensis* ‘Donghong’ genome was used for comparison.

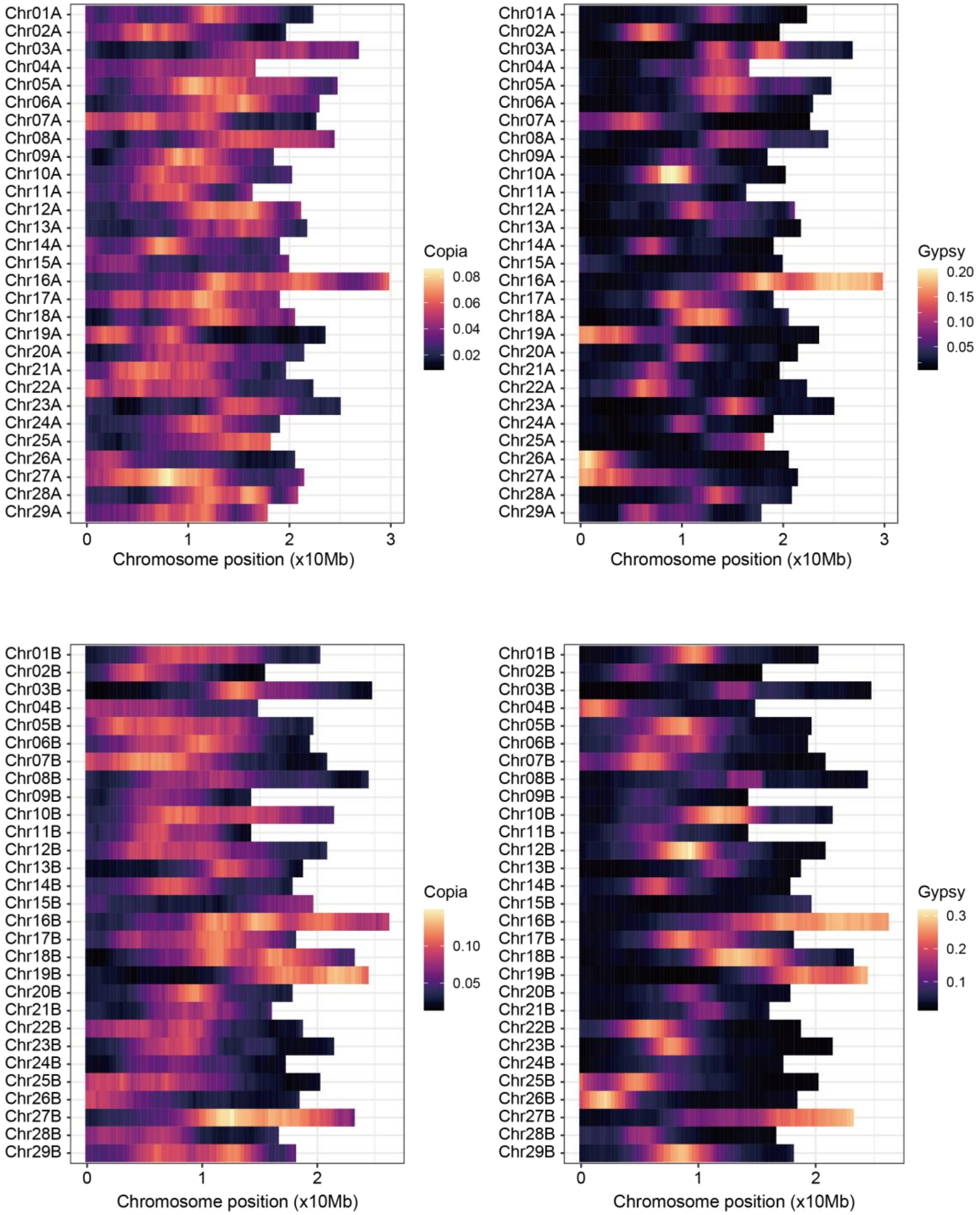

Figure S4. Heatmaps showing the distribution of *Copia* and *Gypsy* LTR-RTs across the chromosomes of *A. valvata*.

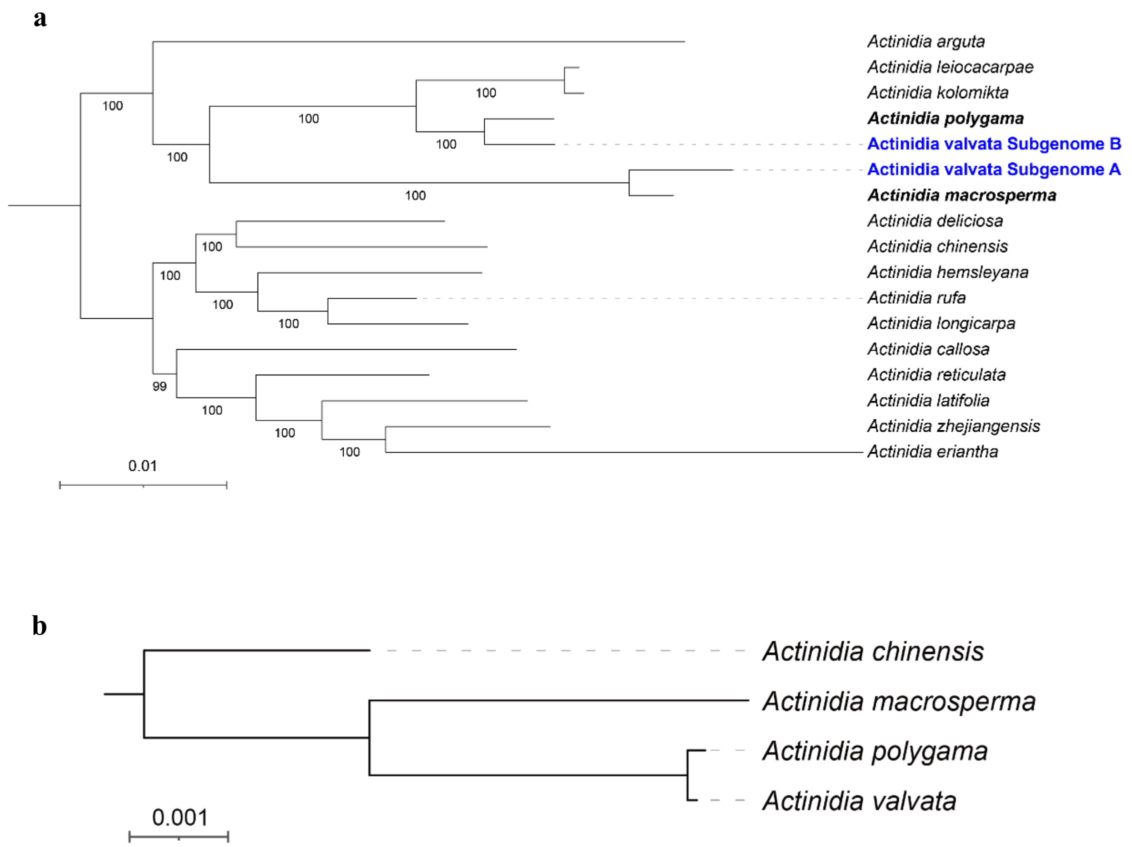

Figure S5. Phylogenetic analysis of Actinidia species. (**a**) Maximum likelihood phylogeny of Actinidia species with published genomes based on 356 orthologs. Numbers on branches represent bootstrap supports. (**b**) Maximum likelihood phylogeny of selected species based on their chloroplast genomes.

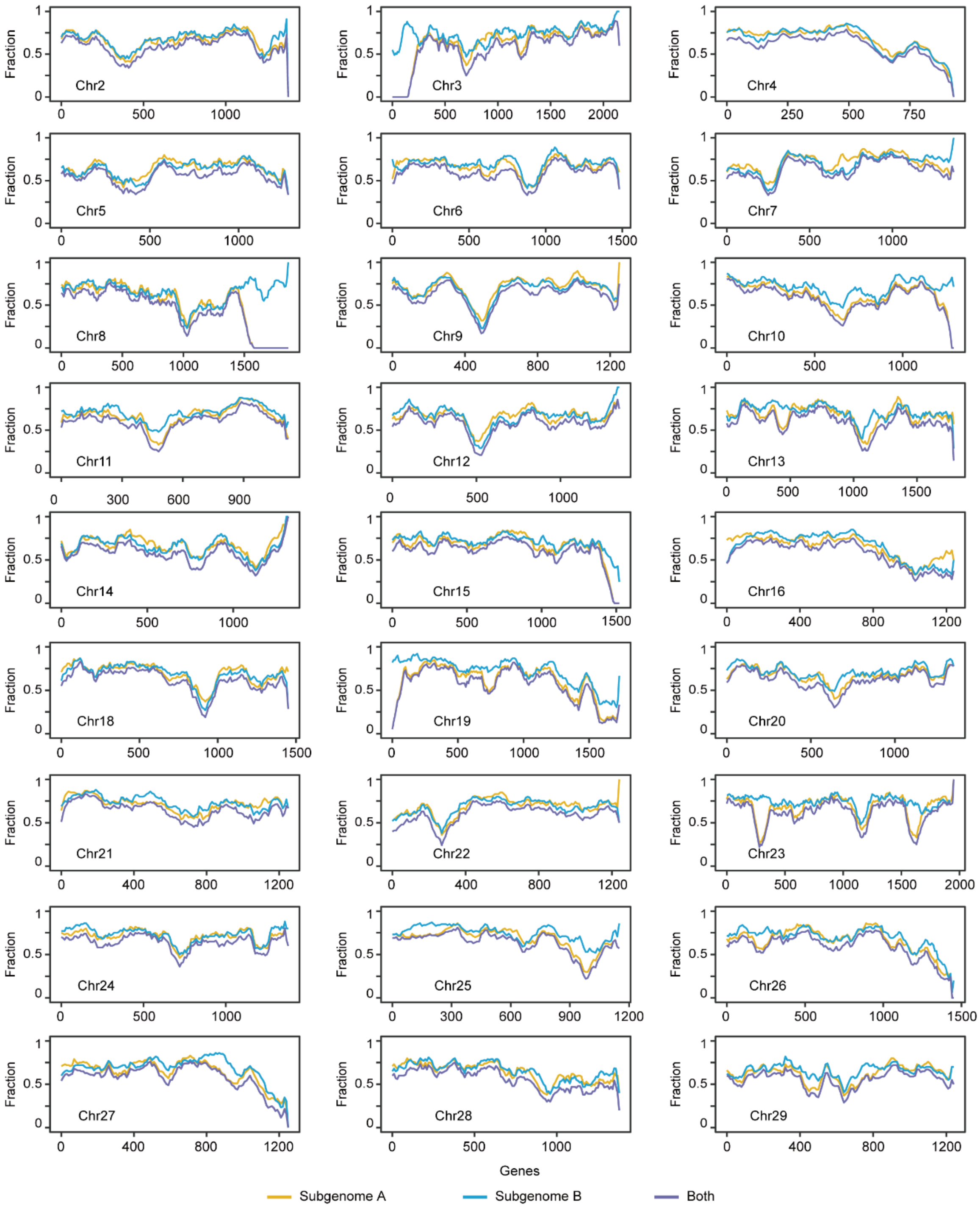

Figure S6. Fractionation in pairs of homoeologous regions in *A. valvata*. Average gene retention rates are presented for 100-gene windows in each subgenome. Retention of genes in subgenomes A, B, and both are depicted with different colored lines.

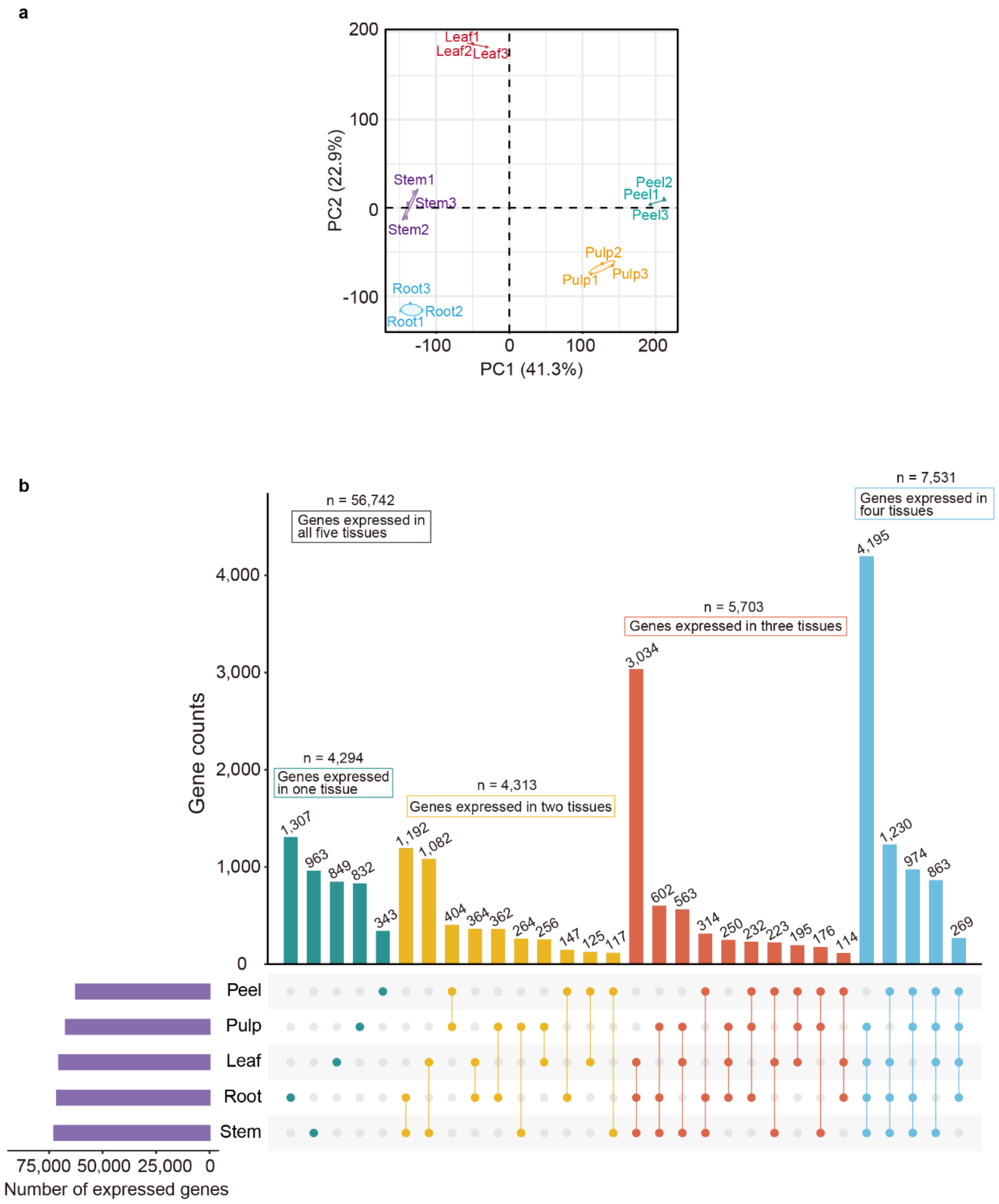

Figure S7. Transcriptome profiling of *A. valvata* in five different tissues. (**a**) PCA analysis of RNA-Seq data from different tissues. (**b**) Comparison of differentially expressed genes across the five tissues.

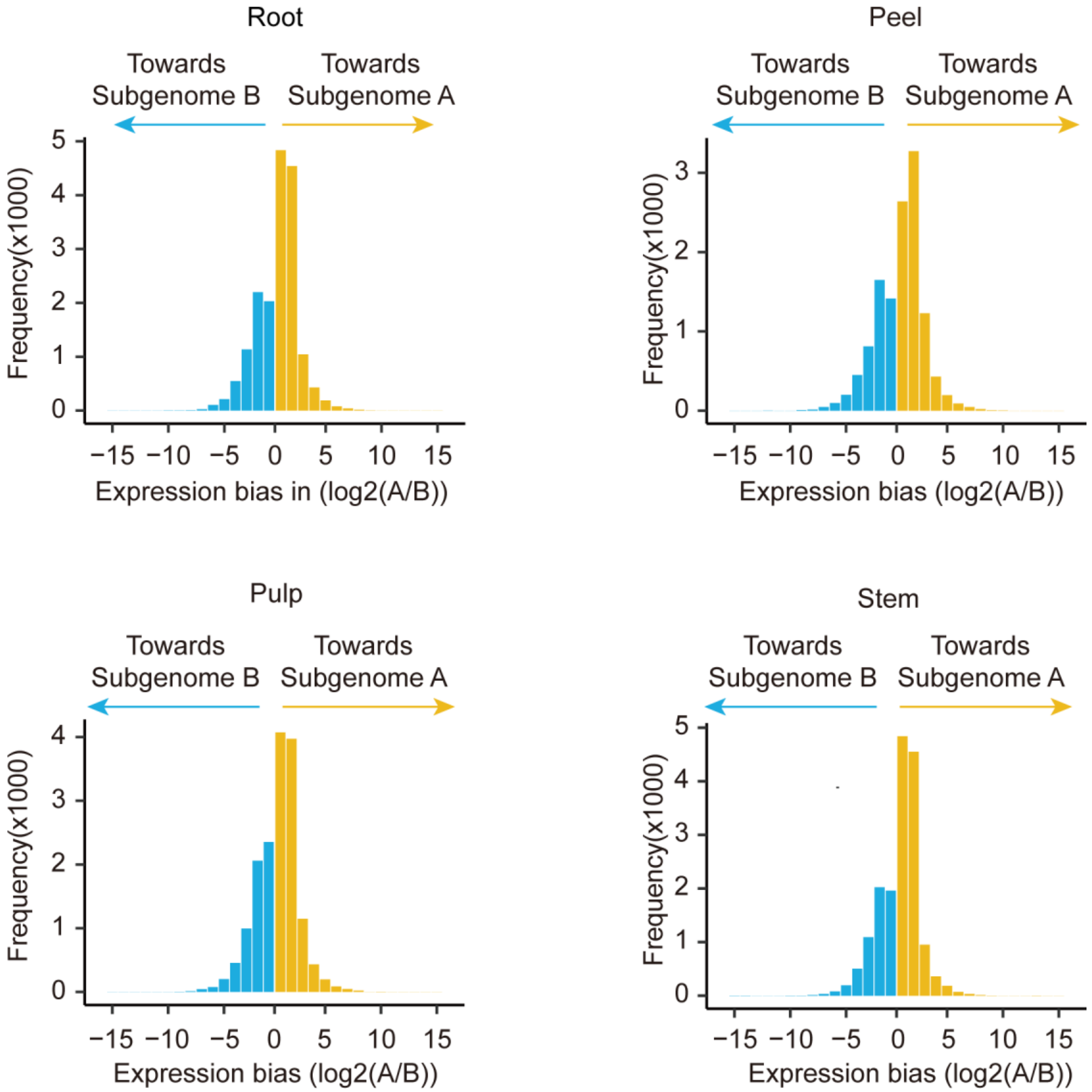

Figure S8. Distribution of homoeolog expression in root, peel, pulp and stem tissues. Only genes with an adjusted *P* value <0.01 are shown.

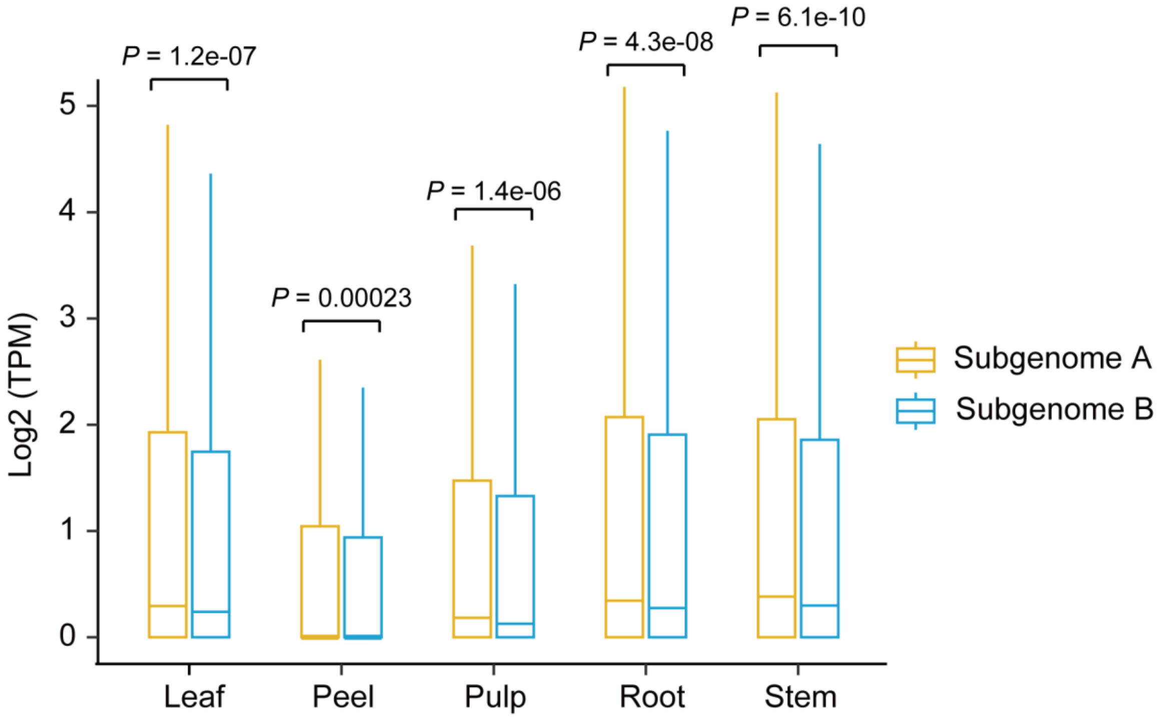

Figure S9. Expression of singleton genes in different tissues of *A. valvata*.

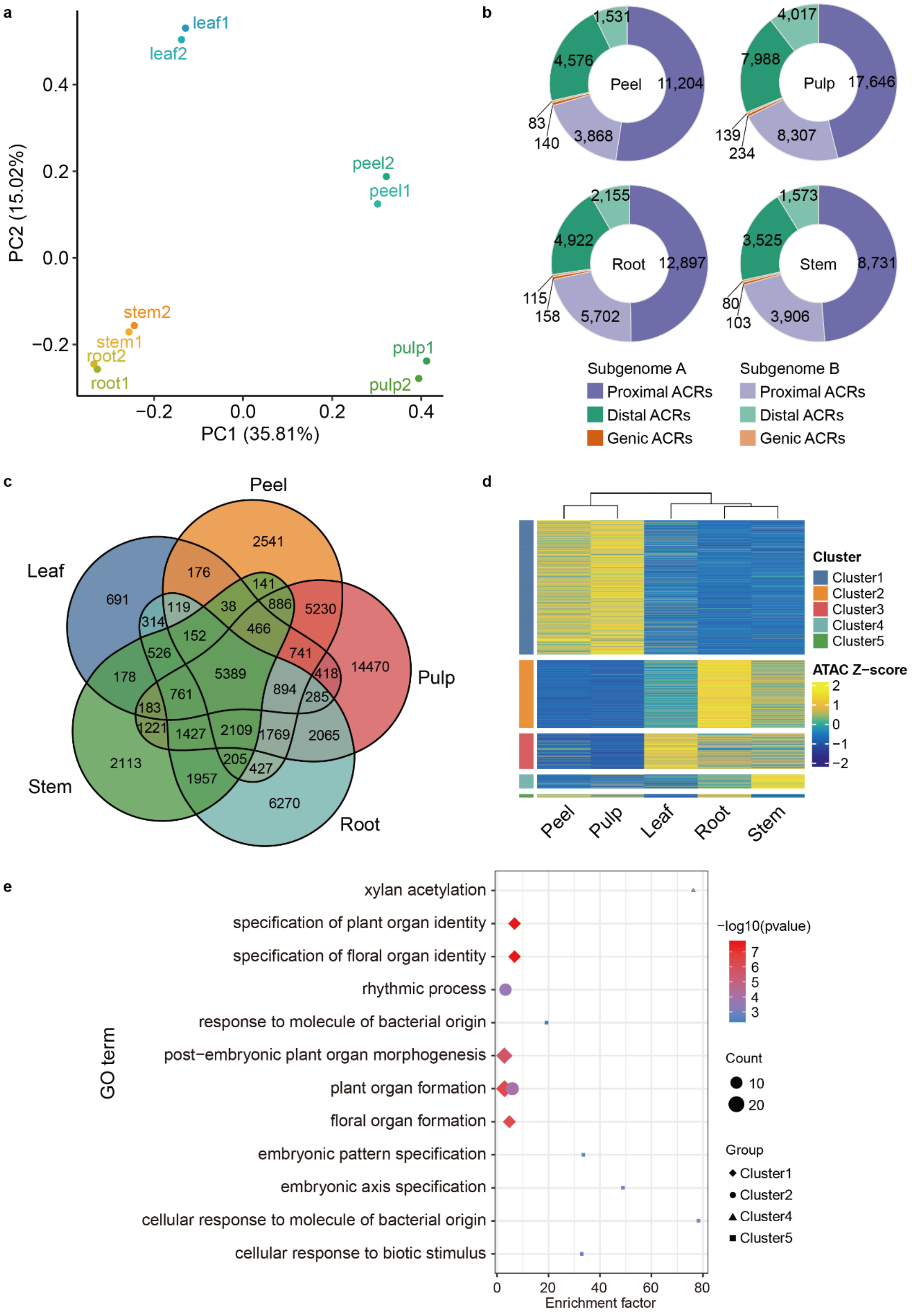

Figure S10. Chromatin accessibility in different tissues of *A. valvata*. (**a**) PCA analysis of ATAC-Seq data from various tissues and biological replicates. (**b**) Distribution of accessible chromatin regions (ACRs) across the *A. valvata* genome. ACRs within 5-kb flanking regions of genes are defined as proximal, while those beyond 5 kb are defined as distal. (**c**) Overlap of ACRs across different tissues. (**d**) Clustering of tissues based on ACRs. (**e**) GO enrichment analysis of genes associated with tissue-specific ACRs.

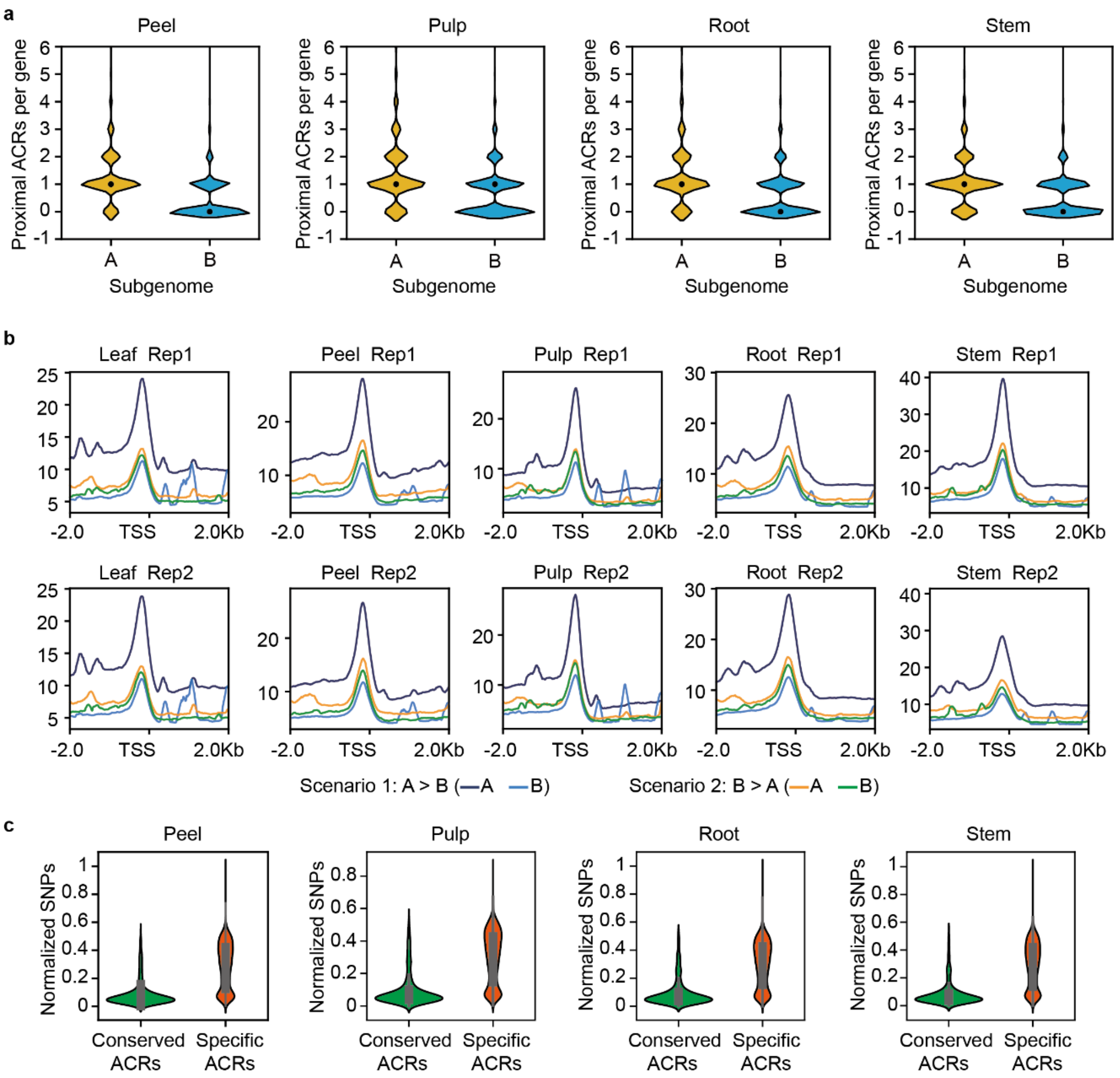

Figure S11. Comparison of ACRs between the two subgenomes of *A. valvata*. (**a**) Distribution of proximal ACRs in the two subgenomes of *A. valvata*. (**b**) Chromatin accessibility of genomic regions containing homoeologs with expression bias. Two scenarios are depicted: one where the subgenome A locus is more highly expressed than the subgenome B locus (A>B), and the other where the subgenome B locus is more highly expressed than the subgenome A locus (B>A). (**c**) Normalized SNP numbers between the two subgenomes in conserved or specific ACRs.

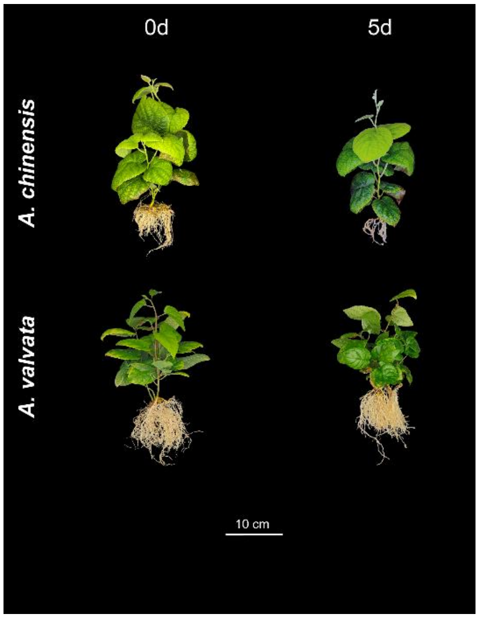

Figure S12. Comparison of waterlogging tolerance of two kiwifruit species, *A. chinensis* and *A. valvata*.

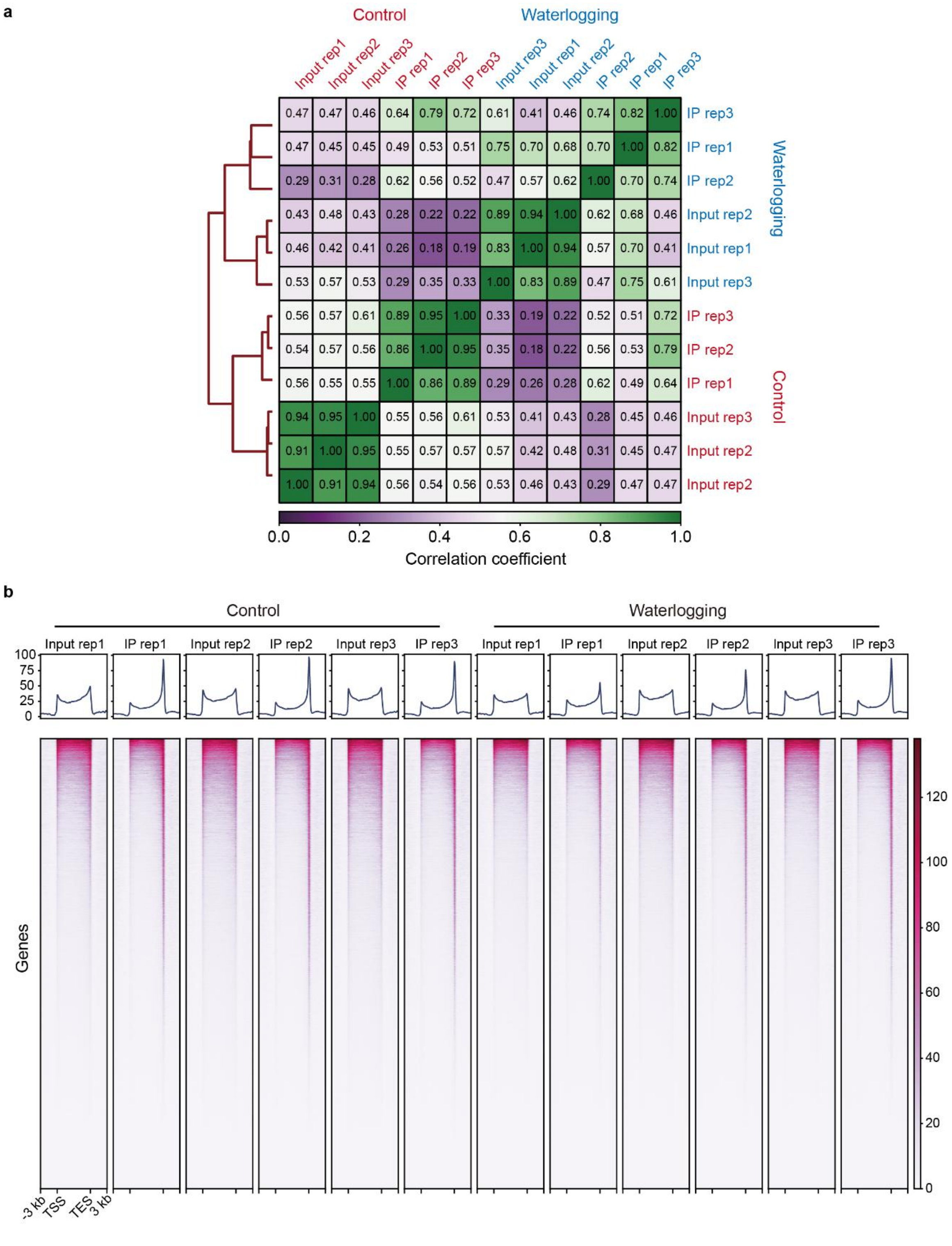

Figure S13. m^{6}A-Seq of *A. valvata*. (**a**) Correlation coefficient of different biological replicates. (**b**) Enrichment of m^{6}A modifications in control and waterlogged samples.

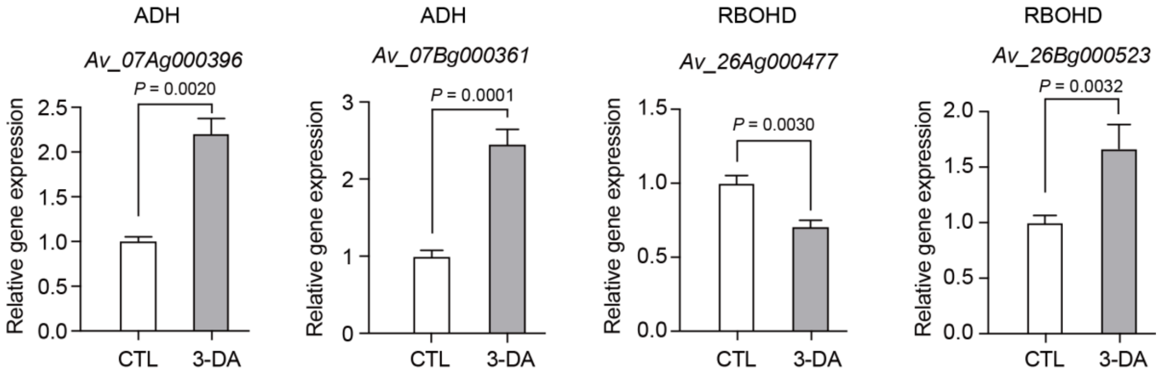

Figure S14. Expression profile of representative waterlogging-responsive genes in *A. valvata* root treated with 3-DA.
